# Acinar ATP8b1/LPC pathway promotes macrophage efferocytosis and clearance of inflammation during chronic pancreatitis development

**DOI:** 10.1038/s41419-022-05322-6

**Published:** 2022-10-22

**Authors:** Wan-jun Yang, Rong-chang Cao, Wang Xiao, Xiao-lou Zhang, Hao Xu, Meng Wang, Zhi-tao Zhou, Huo-ji Chen, Jia Xu, Xue-mei Chen, Jun-ling Zeng, Shu-ji Li, Min Luo, Yan-jiang Han, Xiao-bing Yang, Guo-dong Feng, Yu-heng Lu, Yuan-yuan Ni, Chan-gui Wu, Jun-jie Bai, Zi-qi Yuan, Jin Jin, Guo-wei Zhang

**Affiliations:** 1 Division of Hepatobiliopancreatic Surgery, Department of General Surgery, Nanfang Hospital, Southern Medical University, Guangzhou, China; 2 Nanfang PET Center, Nanfang Hospital, Southern Medical University, Guangzhou, China; 3 Department of the Electronic Microscope Room, Central Laboratory, Southern Medical University, Guangzhou, China; 4 School of Traditional Chinese Medicine, Southern Medical University, Guangzhou, China; 5 Department of Pathophysiology, Southern Medical University, Guangzhou, China; 6 Department of Occupational Health and Medicine, Guangdong Provincial Key Laboratory of Tropical Disease Research, School of Public Health, Southern Medical University, Guangzhou, China; 7 Laboratory Animal Research Center of Nanfang Hospital, Southern Medical University, Guangzhou, China; 8 Guangdong-Hong Kong-Macao Greater Bay Area Center for Brain Science and Brain-Inspired Intelligence, Southern Medical University, Guangzhou, China; 9 Department of Laboratory Medicine, Nanfang Hospital, Southern Medical University, Guangzhou, China; 10 Department of Nuclear Medicine, Nanfang Hospital, Southern Medical University, Guangzhou, China; 11 Division of Nephrology, Nanfang Hospital, Southern Medical University, National Clinical Research Center for Kidney Disease, State Key Laboratory of Organ Failure Research, Guangdong Institute, Guangzhou, China; 12 Southern Medical University, Guangzhou, China; 13 Department of Gynaecology and Obstetrics, Nanfang Hospital, Southern Medical University, Guangzhou, China

**Keywords:** Infectious diseases, Inflammation

## Abstract

Noninflammatory clearance of dying cells by professional phagocytes, termed efferocytosis, is fundamental in both homeostasis and inflammatory fibrosis disease but has not been confirmed to occur in chronic pancreatitis (CP). Here, we investigated whether efferocytosis constitutes a novel regulatory target in CP and its mechanisms. PRSS1 transgenic (*PRSS1*^*Tg*^) mice were treated with caerulein to mimic CP development. Phospholipid metabolite profiling and epigenetic assays were performed with *PRSS1*^*Tg*^ CP models. The potential functions of Atp8b1 in CP model were clarified using Atp8b1-overexpressing adeno-associated virus, immunofluorescence, enzyme-linked immunosorbent assay(ELISA), and lipid metabolomic approaches. ATAC-seq combined with RNA-seq was then used to identify transcription factors binding to the Atp8b1 promoter, and ChIP-qPCR and luciferase assays were used to confirm that the identified transcription factor bound to the Atp8b1 promoter, and to identify the specific binding site. Flow cytometry was performed to analyze the proportion of pancreatic macrophages. Decreased efferocytosis with aggravated inflammation was identified in CP. The lysophosphatidylcholine (LPC) pathway was the most obviously dysregulated phospholipid pathway, and LPC and Atp8b1 expression gradually decreased during CP development. H3K27me3 ChIP-seq showed that increased Atp8b1 promoter methylation led to transcriptional inhibition. Atp8b1 complementation substantially increased the LPC concentration and improved CP outcomes. Bhlha15 was identified as a transcription factor that binds to the Atp8b1 promoter and regulates phospholipid metabolism. Our study indicates that the acinar Atp8b1/LPC pathway acts as an important “find-me” signal for macrophages and plays a protective role in CP, with Atp8b1 transcription promoted by the acinar cell-specific transcription factor Bhlha15. Bhlha15, Atp8b1, and LPC could be clinically translated into valuable therapeutic targets to overcome the limitations of current CP therapies.

## Introduction

The prevalence of chronic pancreatitis (CP) was 42 to 73 per 100,000 adults in the United States between 2001 and 2013, and 36 to 125 per 100,000 people in China, Japan, and India [1, 2]. Clinically, CP not only leads to varying degrees of exocrine and endocrine dysfunction, but also may progress to pancreatic cancer, especially in patients with hereditary CP [3, 4]. Early treatment of CP can prevent these events. However, due to the unknown molecular mechanism of pancreatic tissue fibrosis, there is still no effective treatment.

CP is a chronic inflammatory and fibrotic disease with defective tissue homeostasis linked to genetic, environmental, and other risk factors [5]. Apoptosis governs the main way of acinar death in CP [6, 7]. Engulfment of dying cells by efferocytic macrophages requires recognition of the former by the latter and the formation of the engulfment synapse, which is regulated by a network of “find-me”, “eat-me” and bridging molecules; “don’t eat-me” signals; and specialized phagocytic receptors [8]. Efferocytosis not only serves as a waste disposal mechanism (clearance of dying cells) but also promotes a pro-resolving phenotype in efferocytic macrophages and thereby terminates inflammation. Thus, impaired efferocytosis can lead to chronic inflammation and the development of inflammatory disorders, such as atherosclerosis and autoimmune diseases [8]. Previous studies on efferocytosis have focused mainly on its effector stage, especially the macrophages polarization and function of M2a macrophage; meanwhile, little is known about the “find-me” signals of apoptotic cells. Dying cells can expose and secrete signals that attract phagocytes, favor their engulfment, or promote a return to tissue homeostasis depending on their mode of death. Different forms of cell death can also induce either pro-inflammatory or anti-inflammatory signaling by modulating macrophage activity following efferocytosis [9]. However, to date, the existence of efferocytosis in CP and its influence on CP progression have not been reported.

Activated macrophages are usually classified as M1-like or M2-like macrophages, which play different roles in inflammation. M1 macrophages, termed inflammatory macrophages, are intimately involved in the pro-inflammatory response and produce various inflammatory-related factors, such as IL-6, IL-12, and TNF. In contrast, M2 macrophages usually promote the anti-inflammatory response and produce anti-inflammatory factors such as TGF-β [10]. M1 macrophages express CD86, CD80, and CD16/32, while M2 macrophages overexpress arginase-1 (ARG1), mannose receptor (CD206), anti-inflammatory factors (e.g, IL-10), and the chemokines CCL17 and CCL22. During the early stage of inflammation, macrophages usually polarize into the M1 phenotype to defend against pathogens by releasing inflammatory factors and recruiting inflammatory cells. Subsequently, macrophages are polarized into the M2a phenotype to release anti-inflammatory factors and repair damaged tissue [11].

Sphingolipids represent a large class of lipids playing diverse functions in various physiological and pathological processes. Sphingomyelin is the most abundant sphingolipids in the cell, is an essential part of plasma membrane [12]. The metabolites of sphingomyelin, such as ceramide (Cer), sphingosine (Sph), and sphingosine 1-phosphate (S1p) [13], were closely associated with the regulation of cell proliferation and apoptosis. In the process of efferocytosis, apoptotic cells induced efferocytic immune cells, like macrophage, by releasing chemokines, lipids (S1p and lysophosphatidylcholine (LPC)). And then, macrophages engage apoptotic cells through cell-surface receptors that directly bind molecules on the apoptotic cells surface, which was known as “find-me” signal [9].

In this study, we first confirmed the occurrence of phospholipid metabolism dysregulation and defective efferocytosis in *PRSS1* transgenic (*PRSS1*^*Tg*^) CP mice. Next, we observed that the level of an important “find-me” signal LPC, a phospholipid present at different locations in the plasma membrane [14], decreased along with ATPase class I type 8b member 1 (Atp8b1) during CP development. In addition, increased methylation in the Atp8b1 promoter region led to transcriptional inhibition. Atp8b1 functions as a phospholipid flippase located in the membrane, and is widely distributed in the lung [15], pancreas [16], and other organs and tissues [17]. Meanwhile, *PRSS1*^*Tg*^ CP mice, which displayed decreased Atp8b1 and LPC levels, exhibited decreased acinar apoptosis and M2a macrophage infiltration. Bhlha15, an acinar-specific transcription factor (TF), was identified as a regulatory partner of Atp8b1 that protects against CP pathogenesis. These data not only identify existence of defective efferocytosis in CP, but also reveal that Bhlha15/Atp8b1/LPC pathway plays a protective role in the disease, which suggests a potential therapeutic approach for CP.

## Results

### Defective efferocytosis and dysregulated phospholipids metabolism are involved in the fibrotic response in CP

**Fig. 1:**
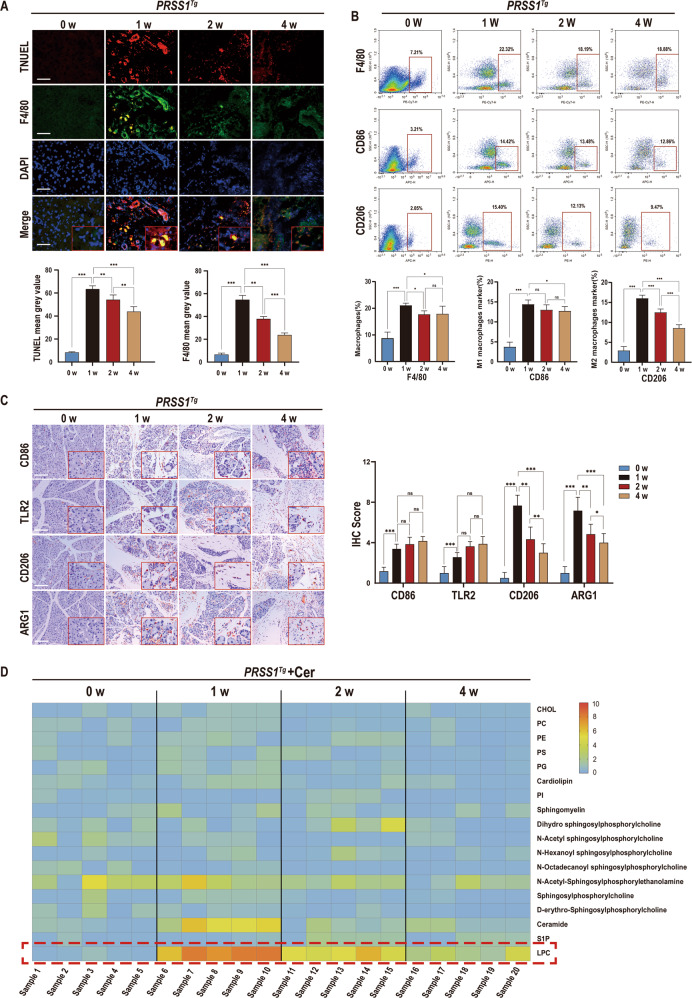
Identification of impaired efferocytosis and dysregulated phospholipid metabolism in chronic pancreatitis(CP). To establish a caerulein-induced CP model, *PRSS1*^*Tg*^ mice were intraperitoneally injected with 15 μg/ml caerulein dissolved in phosphate-buffered saline at a dose of 50 μg/kg each hour for 8 hours, twice per week, for a total of four weeks. **A** TUNEL staining to show apoptosis were performed and immunofluorescence staining was to detect the expression of F4/80 in pancreatic tissues from caerulein-treated *PRSS1*^*Tg*^ mice at weeks 0, 1, 2, and 4. DAPI staining to show nucleus was performed. The mean gray values were used to quantify the expression of F4/80 and the degree of apoptosis. **B** Flow cytometry was performed to analyze the proportion of macrophages maker(F4/80+PE-CY7), M1-like macrophages marker(CD86 + APC), and M2-like macrophages marker(CD206 + PE) in the pancreatic tissues of caerulein-treated *PRSS1*^*Tg*^ mice at weeks 0, 1, 2, and 4. **C** The expression levels of M1 macrophage markers (CD86 and TRL2) and M2a macrophage markers (CD206 and ARG1) in pancreatic tissues from caerulein-treated *PRSS1*^*T*g^ mice were measured by immunohistochemistry, and immunohistochemical scoring was performed. **D** The levels of phospholipid metabolites were assessed by liquid chromatography-tandem mass spectrometry (LC–MS/MS), orange represented high expression, blue mean low expression. The data are presented as the means ± SDs. Three biological replicates were performed. Significant differences between two groups were analyzed by Student’s t test, and one-way analysis of variation was performed to investigate the differences among more than two groups. ns no significant difference; *p ≤ 0.05; **p ≤ 0.01; ***p ≤ 0.001. Scale bars = 100 µm.

To investigate whether defective efferocytosis exist during CP progression, *PRSS1*^*Tg*^ mice were administered different treatment schedules of caerulein to induce CP as reported previously. Pancreatic acinar cell apoptosis was increased in the early stage of CP development, peaking in week 1, and then decreased over the remaining three weeks of the experiment (Fig. 1A). Furthermore, substantial collagen deposition and morphological changes in the pancreas were observed in CP tissue compared with 0-week from *PRSS1*^*Tg*^ mice (Fig. S1A).

Macrophages are usually divided into two categories, M1-like and M2-like macrophages, The major cells mainly responsible for apoptotic cell recognition and clearance in vivo are M2a subtypes [18], which prompted us to investigate temporo-spatial changes in macrophage infiltrate. The expression level of F4/80, a key marker of macrophages, markedly increased at 1 week, but decreased during CP progression (Fig. 1A and S1E). It indicated that the level of macrophage infiltration changed obviously. Flow cytometry analysis indicated that the proportion of pancreatic macrophages were achieved to the highest level at 1 week but decreased in the following stages. In addition, a significant increase in alpha-smooth muscle actin (α-SMA) expression over time (Fig. S1D) indicated that pancreatic parenchymal fibrosis increased after CP induction. M1 (CD86 and TLR2) and M2a (CD206 and ARG1) macrophage markers expression in CP tissues from *PRSS1*^*Tg*^ mice were further assessed. Specifically, M1 macrophage marker expression was increased in CP tissues compared with 0-week from *PRSS1*^*Tg*^ mice, but this expression did not significantly differ among week 1, week 2, and week 4. In contrast, M2a macrophage marker expression peaked at week 1 and then decreased gradually after model induction (Fig. 1C), the flow cytometric analysis indicated that the proportion of M2a macrophages reached the highest level at 1 week, but gradually reduced along with the aggravation of fibrosis (Fig. 1B). Additionally, the levels of the inflammatory factors interleukin IL-1β, IL-6, and tumor necrosis factor alpha (TNF-α) were significantly elevated in 1, 2, 4-week groups compared with 0-week group (Fig. S1B).

Because modified membrane lipids, especially phospholipids pathway, accounts for the most important apoptosis-specific “find-me” signals of efferocytosis [19, 20], we next examined 18 selected phospholipid metabolites. In caerulein-treated *PRSS1*^*Tg*^ mice at 0, 1, 2, 4-week, the levels of 4 phospholipid-related metabolites (n-acetyl-sphingosylphosphorylethanolamine, dihydrosphingosylphosphorylcholine, ceramide, and LPC) were significantly altered (Fig. 1D). The level of an important “find-me” signal-lysophosphatidylcholine (LPC), a phospholipid present at different locations in the plasma membrane, peaked in week 1 and then decreased over time after CP induction (Fig. 1D).

Collectively, above results showed that the quantity of macrophages, especially M2a macrophages which play an important role in engulfment of dying cells, decreased from week 1 to week 4 in *PRSS1*^*Tg*^ CP model. Meanwhile, abundance of LPC, an important “find me” signal from apoptotic acinar cells, also decreased over time. These data indicate that defective efferocytosis and dysregulated phospholipid metabolism exist in CP and that changes in the LPC concentration produced via the phospholipid pathway are probably associated with the pathogenesis of CP.

### Atp8b1 is downregulated in pancreatic acinar cells during CP development

**Fig. 2:**
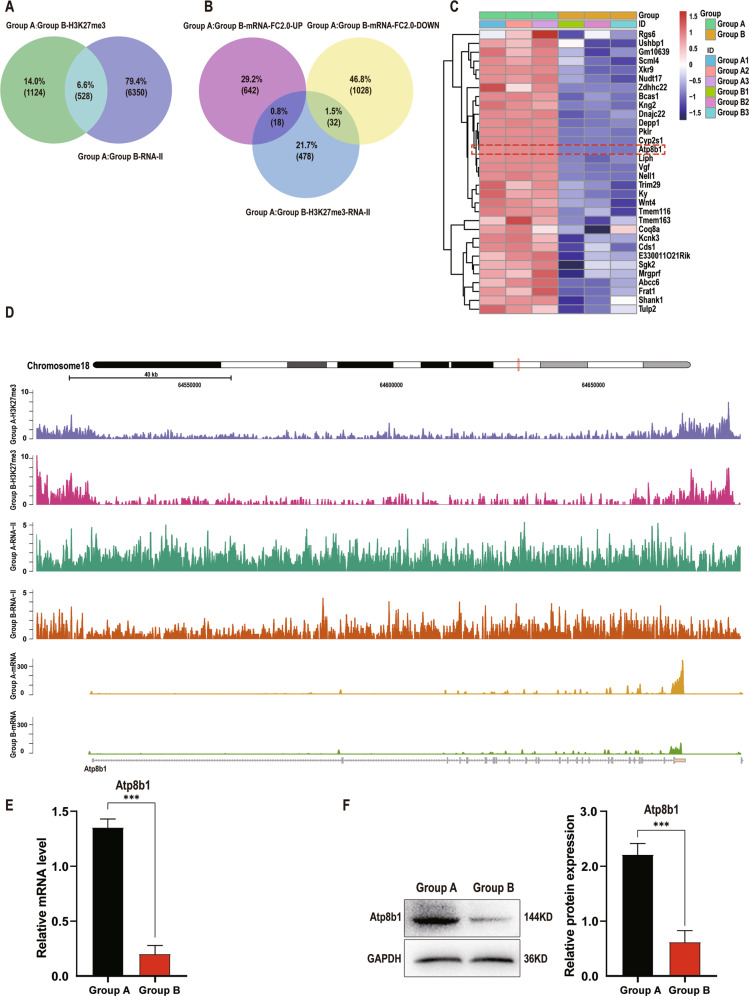
Atp8b1 is downregulated in CP tissues from *PRSS1*^*Tg*^ mice. H3K27me3 ChIP-seq, RNA pol II ChIP-seq and mRNA-seq analyses of pancreatic tissues from Group A (treated with caerulein for 1 week) and Group B (treated with caerulein for 4 weeks) were applied to identify genes that potentially regulate phospholipid metabolism. **A** Venn diagram analysis of the overlap between the H3K27me3 ChIP-seq and RNA pol II ChIP-seq. **B** Venn diagram analysis of the overlap between the above 528 genes and differentially expressed mRNAs in Group A and Group B separately. **C** Heatmap of the 32 downregulated genes. **D** Signal tracks showing the binding and co-occupancy of H3K27me3 and RNA pol II at the Atp8b1 gene locus and mRNA-seq signals for genes in pancreatic acinar cells in Groups A and B. Purple, Group A-H3K27me3; Red, Group B-H3K27me3, Green, Group A- RNA pol II; Orange, Group B- RNA pol II; Yellow, Group A-mRNA; Cyan-blue, Group B- mRNA. **E** The total Atp8b1 mRNA and **F** protein expression levels in pancreatic tissue in Group A and B were measured by quantitative RT-PCR and western blotting, respectively. The results were measured by densitometry and expressed by expression relative to GAPDH as the reference protein. The data are presented as the means ± SDs; *n* = 3 biological replicates. ***p ≤ 0.001.

To clarify potential genes responsible for phospholipid metabolism in CP, epigenetic assays and screening are performed on pancreatic tissues of *PRSS1*^*Tg*^ mice that were treated with caerulein for 4 weeks (Group B) and those treated with caerulein for 1 week (Group A). RNA-seq and H3K27me3 ChIP-seq results were used to compare Group A and Group B and 528 genes were found to be differentially expressed in both datasets (Fig. 2A), among which 32 differentially expressed genes were identified as downregulated by RNA-seq analysis (Fig. 2B). We then searched protein databases (UniProt and STRING) for gene products specifically related to phospholipid metabolism and identified Atp8b1 as a potential regulator that contributes to the dysregulation of phospholipid metabolism in CP pathogenesis (Fig. 2C). Atp8b1 was identified by histone 3 lysine 27 trimethylation (H3K27me3) ChIP-seq, RNA pol II ChIP-seq and RNA-seq analyses of pancreatic acinar cells from *PRSS1*^*Tg*^ mice (Fig. 2D) qPCR (Fig. 2E) and Western blotting (Fig. 2F) analysis revealed that the increase in mRNA and protein expression of Atp8b1 in Group A was attenuated in Group B.

**Fig. 3:**
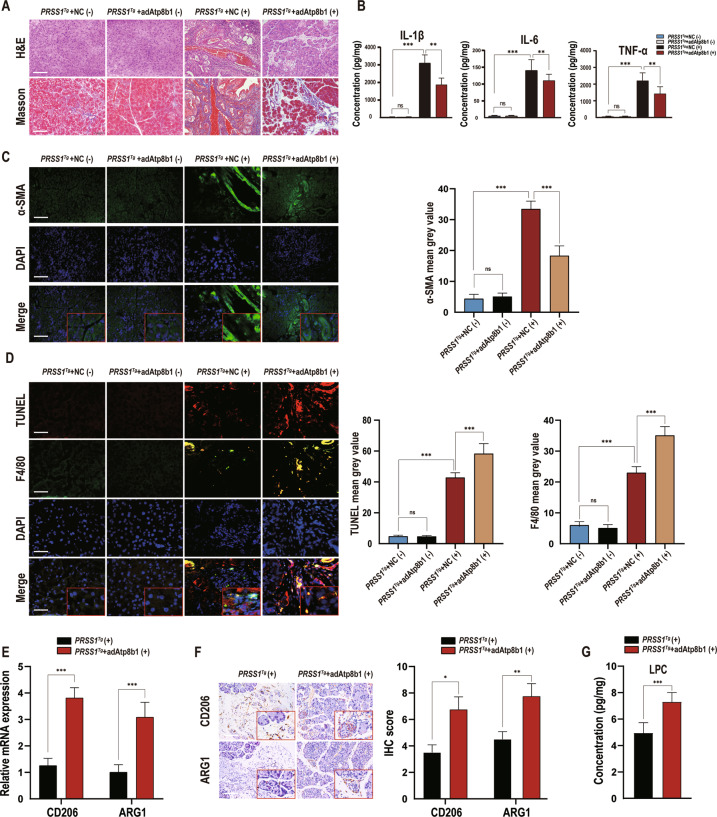
Atp8b1 reverses the impairment of efferocytosis and dysregulation of phospholipid metabolism in acinar cells during CP development. AdAtp8b1 was used to induce Atp8b1 overexpression in the pancreas of *PRSS1*^*Tg*^ mice. The pancreases of *PRSS1*^*Tg*^ mice were infected with adenoviral vectors harbouring a scrambled adRNA for the negative control (NC). **A** Structural alterations and fibrosis in pancreatic tissues from adAtp8b1 and NC groups *PRSS1*^*Tg*^ mice treated (+) or untreated (−) with caerulein were evaluated by H&E staining and Masson’s trichrome staining, respectively. **B** The levels of IL-1β, IL-6, and TNF-α in pancreatic tissues from caerulein-treated and untreated *PRSS1*^*Tg*^ mice were quantitatively analyzed by ELISA. **C** α-SMA expression in pancreatic tissues from caerulein-treated and untreated *PRSS1*^*Tg*^ mice was detected by immunofluorescence staining, the mean gray values calculated by ImageJ were used to quantitatively analyze the degree of fibrosis. The mean gray values was calculated using integrated density divided by area. **D** Apoptosis and F4/80 expression were detected by a TUNEL assay and immunofluorescence staining, respectively, in pancreatic tissues from caerulein-treated and untreated *PRSS1*^*Tg*^ mice. The mean gray values calculated by ImageJ were used to quantify these parameters. **E** The mRNA expression of CD206 and ARG1 in the pancreatic tissues from adAtp8b1-treated and untreated *PRSS1*^*T*g^ CP mice was measured by RT-PCR. **F** Immunohistochemical staining was used to detect CD206 and ARG1 expression in pancreatic tissues from adAtp8b1-treated and untreated *PRSS1*^*T*g^ CP mice. **G** The concentration of LPC in pancreatic tissues from adAtp8b1-treated and untreated *PRSS1*^*T*g^ CP mice was assessed by LC–MS/MS. *PRSS1*^*Tg*^ (+) and *PRSS1*^*Tg*^ (−) represented mice treated and untreated with caerulein, respectively. The data are presented as the means ± SDs. ns no significant difference; *p ≤ 0.05; **p ≤ 0.01; ***p ≤ 0.001. Scale bars = 100 µm.

To confirm the association of Atp8b1 downregulation with CP, Atp8b1 expression in the pancreas was complemented using an Atp8b1-overexpressing adeno-associated virus (AAV; *adAtp8b1*) (Fig. S3A); qRT-PCR and Western blotting for Atp8b1 expression confirmed a high transduction efficiency (Fig. S3B, C). Notably, the caerulein-induced fibrosis and histologic changes in the pancreas in *PRSS1*^*Tg*^ mice were alleviated upon restoration of Atp8b1 expression (Fig. 3A, C). Moreover, the levels of IL-1β, IL-6, and TNF-α were significantly inhibited after Atp8b1 expression was restored (Fig. 3B). Furthermore, *adAtp8b1*-injected *PRSS1*^*Tg*^ mice exhibited a higher F4/80 level (Fig. 3D) and LPC concentration (Fig. 3G) than negative control AAV-injected mice in pancreatic tissues, as well as increased expression of M2a macrophage markers (CD206 and ARG1) (Fig. 3E, F). These results suggest that Atp8b1 expression is downregulated in CP and that restoration of Atp8b1 expression can significantly improve CP outcomes.

### Atp8b1 increases acinar LPC concentration, promoting M2a macrophage efferocytosis of apoptotic acinar cells in CP

**Fig. 4:**
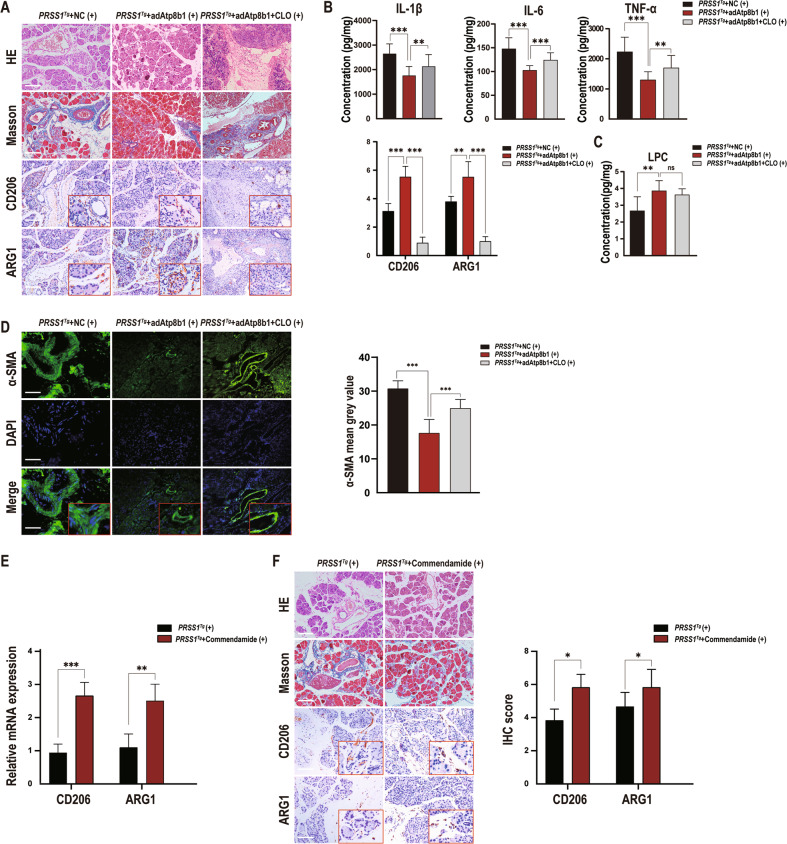
LPC plays a role in promoting efferocytosis by binding to its receptor G2A. CLO was used for macrophage knockout in adAtp8b1 *PRSS1*^*T*g^ mice treated with careulein. **A** Structural alterations, fibrosis, and the expression of CD206 and ARG1 were evaluated by H&E staining, Masson’s trichrome staining, and immunohistochemical staining, respectively, of pancreatic tissues from *PRSS1*^*T*g^ mice treated with caerulein in the adAtp8b1 and macrophage knockout groups. **B** Quantitative expression of IL-1β, IL-6, and TNF-α in pancreatic tissues from caerulein-treated *PRSS1*^*Tg*^ mice. **C** The concentration of LPC in pancreatic tissues from caerulein-treated *PRSS1*^*Tg*^ mice was measured by LC–MS/MS. **D** α-SMA expression detected by immunofluorescence staining was used to analyze the degree of fibrosis from *PRSS1*^*T*g^ mice treated with caerulein in the adAtp8b1 and macrophage knockout groups. The mean gray values were used to quantitatively analyze the degree of fibrosis. **E** The mRNA expression of CD206 and ARG1 in the pancreatic tissues from adAtp8b1-treated and untreated *PRSS1*^*T*g^ CP mice was measured by RT-PCR. **F** Structural alterations, fibrosis, and the expression of CD206 and ARG1 were evaluated by H&E staining, Masson’s trichrome staining, and immunohistochemical staining, respectively, of pancreatic tissues from CP model mice treated with or without commendamide (a G2A agonist). The data are presented as the means ± SDs. ns no significant difference; *p ≤ 0.05; **p ≤ 0.01; ***p ≤ 0.001. Scale bars = 100 µm.

To further investigate the mechanism by which Atp8b1 is involved in CP pathogenesis, *PRSS1*^*Tg*^ mice were treated with CLOs to deplete macrophages after injection of adAtp8b1 and then treated with caerulein to induce CP; inflammation severity was then assessed by histological examination. Substantial fibrosis, histologic changes, increased inflammatory factor expression and decreased M2a macrophage marker expression were observed in CLO-treated *PRSS1*^*Tg*^ + *adAtp8b1* mice compared with non-CLO-treated *PRSS1*^*Tg*^ + *adAtp8b1* mice (Fig. 4A, B, D). Additionally, LPC concentration was significantly elevated in CLO-treated *PRSS1*^*Tg*^ + *adAtp8b1* mice compared to *PRSS1*^*Tg*^ + NC AAV mice but not significantly different between the CLO-treated *PRSS1*^*Tg*^ + *adAtp8b1* mice and non-CLO-treated *PRSS1*^*Tg*^ + *adAtp8b1* mice (Fig. 4C). These results reveal that overexpression of Atp8b1 increased the LPC concentration and reversed the impairment of efferocytosis and dysregulation of acinar phospholipid metabolism in *PRSS1*^*Tg*^ CP mice, confirming Atp8b1 as protective for CP pathogenesis. In addition, macrophage-depleted *PRSS1*^*Tg*^ mice failed to clear apoptotic acinar cells and developed fibrotic lesions histologically, regardless LPC abundance was elevated or not, which further reveal that macrophage-related efferocytosis is involved in CP pathogenesis.

To ascertain the role of LPC in CP development, *PRSS1*^*Tg*^ mice were treated with the G2A receptor agonist commendamide, followed by assessment of H&E staining and Masson’s trichrome staining. The increases in substantial pancreatic impairment and collagen expression observed in *PRSS1*^*Tg*^ CP mice were significantly ameliorated upon treated with commendamide (Fig. 4F). CD206 and ARG1 expression levels were increased in *PRSS1*^*Tg*^ mice CP model in the commendamide-treated group compared with those in the untreated group (Fig. 4E, F), which indicate that activation of LPC receptor G2A relieves fibrotic response by triggering macrophage-related efferocytosis.

Collectively, these results indicate that Atp8b1 promotes efferocytosis through the binding of LPC to its receptor G2A.

### Bhlha15 is a regulatory binding transcription factor of Atp8b1

**Fig. 5:**
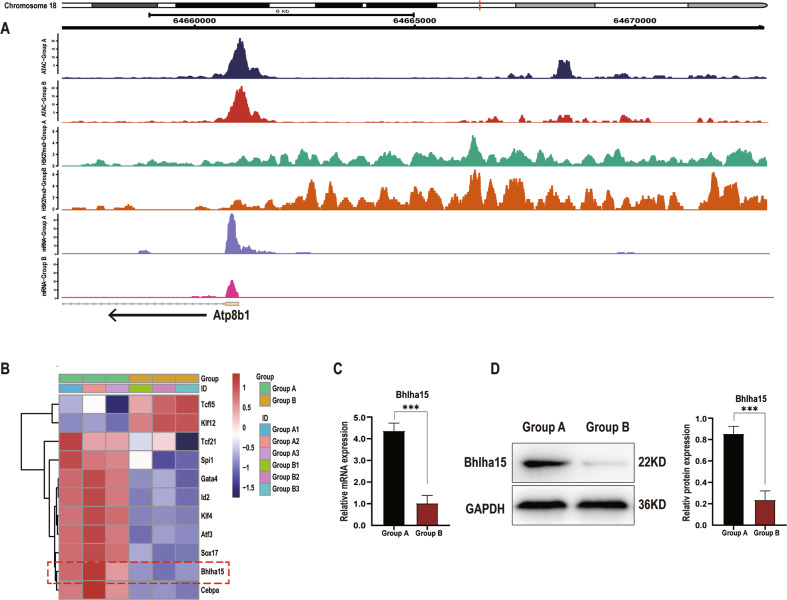
Bhlha15 binds to the promoter of Atp8b1 in acinar cells. **A** Signal tracks showing the open chromatin region in the Atp8b1 promoter, as determined by ATAC-seq, in Group A and B. The differential peaks in the two groups are shown above the tracks. The binding and co-occupancy of H3K27me3 and mRNA-seq signals of genes in pancreatic acinar cells from Groups A and B w ere visualized with Integrative Genomics Viewer. **B** Heatmap shown 11 genes were identified by predicting the binding sites of transcription factor to the open chromatin regions around the TSS of Atp8b1 using Jaspar (threshold score, > 80). The mRNA (**C**) and protein (**D**) expression levels of Bhlha15 in pancreatic tissue were determined by quantitative RT–PCR and western blotting, respectively. ***p ≤ 0.001.

To identify upstream transcription factors binding to Atp8b1, ATAC-seq analysis of pancreatic acinar cells from *PRSS1*^*Tg*^ CP mice was performed. H3K27me3 modification was increased at the Atp8b1 transcription start site (TSS) and Atp8b1 expression was downregulated in Group B compared to Group A (Fig. 5A). By predicting the binding of transcription factors to the open regions around the TSS using Jaspar, we identified 11 transcription factors (Fig. 5B). We then analyzed the transcription factors with the UniProt database to identify those with functions strongly correlated with phospholipid metabolism, and thereby screened out 3 transcription factors (Fig. S4B); we ultimately identified Bhlha15 as a potential upstream transcription factor of Atp8b1. The mRNA and protein expression of Bhlha15 in the pancreatic tissue of *PRSS1*^*Tg*^ mice was significantly downregulated in Group B compared with Group A (Fig. 5C, D).

**Fig. 6:**
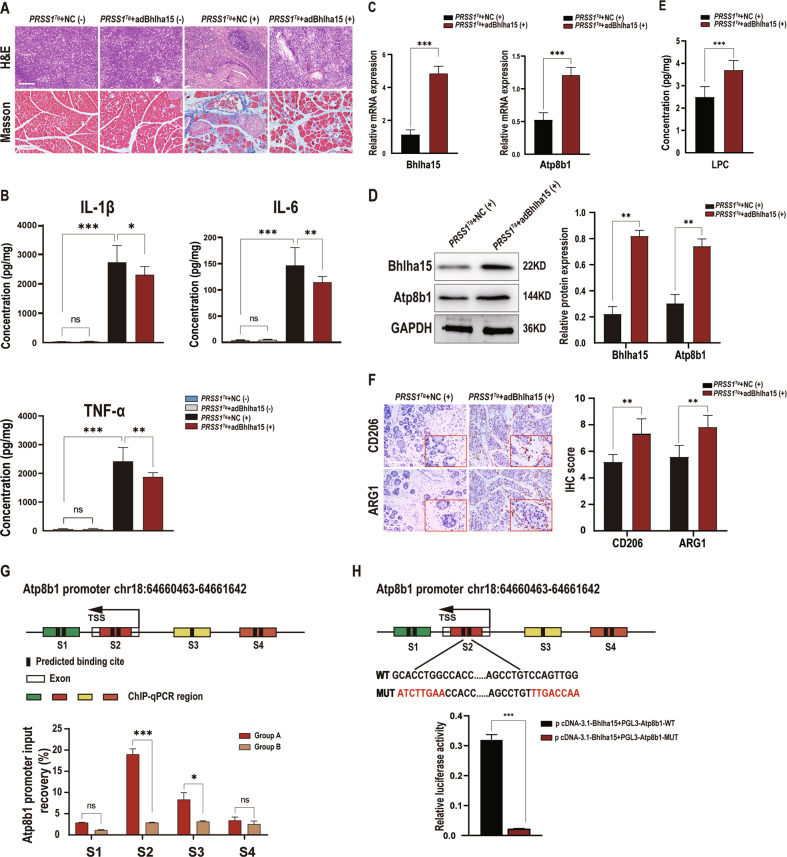
Overexpression of Bhlha15 ameliorates apoptosis and CP progression. AdBhlha15 was used to overexpress Bhlha15 in the pancreas of caerulein-treated or untreated *PRSS1*^*Tg*^ mice. The pancreases of *PRSS1*^*Tg*^ mice were infected with adenoviral vectors harbouring a scrambled adRNA for the negative control (NC). **A** Structural alterations and fibrosis were evaluated by HE staining and Masson’s trichrome staining, respectively, of pancreatic tissues from *PRSS1*^*Tg*^ mice. **B** Quantitative analysis of IL-1β, IL-6, and TNF-α levels in pancreatic tissues from caerulein-treated or untreated *PRSS1*^*Tg*^ mice. **C** The Bhlha15 and Atp8b1 mRNA expression levels in pancreatic tissue from the Bhlha15 overexpression group and negative control group (NC) treated with caerulein were measured by quantitative RT–PCR. **D** The protein expression of Bhlha15 and Atp8b1 in pancreatic tissues from adBhlha15-treated or negative control *PRSS1*^*T*g^ mice treated with caerulein was detected by western blotting and quantified. **E** The concentration of LPC in pancreatic tissues from adBhlha15-treated or untreated *PRSS1*^*T*g^ CP mice was measured by LC–MS/MS. **F** Immunohistochemical staining was performed to detect CD206 and ARG1 expression in pancreatic tissues from *PRSS1*^*T*g^ mice. **G** Binding of Bhlha15 to the Atp8b1 promoter region was assessed in vitro in pancreatic acinar cells from mice in Group B (treated with caerulein for 4 weeks) or Group A (treated with caerulein for 1 week) by ChIP using anti-Bhlha15 antibodies or IgG. Input and immunoprecipitated DNA purified by ChIP were analyzed using quantitative RT–PCR, relative mRNA levels were calculated using the comparative ΔΔCt method and presented as a percentage of the controls. mRNA levels of GAPDH genes were used as controls for normalization. The predicted Bhlha15 binding sites are marked S1, S2, S3, and S4. **H** The luciferase assay indicated that the S2 region of the Atp8b1 promoter was the target binding site for Bhlha15. A relative firefly luciferase/ Renilla luciferase of each samples were calculated, then mean firefly luciferase/Renilla luciferase ratio of biological replicates were subsequently calculated. To obtain fold enrichment of each group compared to NC group, NC group was designed as normalization, The red text indicates the mutant binding sites. The data are presented as the means ± SDs. ns no significant difference; *p ≤ 0.05; **p ≤ 0.01; ***p ≤ 0.001.

To investigate the role of Bhlha15 in CP development, we used a Bhlha15-overexpressing AAV for pancreatic transduction in caerulein-treated *PRSS1*^*Tg*^ mice and then assessed pathological changes by H&E staining and Masson’s trichrome staining. Inflammatory cell infiltration, collagen deposition and inflammatory cytokine levels were decreased in *PRSS1*^*Tg*^ CP mice upon Bhlha15 overexpression (Fig. 6A, B), indicating disease amelioration. Furthermore, the mRNA and protein levels of Bhlha15 and Atp8b1 were significantly increased in Bhlha15-overexpressing *PRSS1*^*Tg*^ CP mice compared with *PRSS1*^*Tg*^ + NC AAV CP mice (Fig. 6C, D). The LPC concentration was also examined in these groups, revealing a significant increase in the Bhlha15 overexpression group (Fig. 6E). Moreover, the expression of M2a macrophage markers in *PRSS1*^*Tg*^ CP mice was enhanced upon Bhlha15 overexpression (Fig. 6F).

Next, ChIP with quantitative PCR (ChIP-qPCR) (Fig. 6G) was conducted to verify the Jaspar prediction that two potential binding sites for Bhlha15 are located in the Atp8b1 promoter. The ChIP assay showed substantial increases in the binding of Bhlha15 to chromatin in the S2 and S3 regions of the Atp8b1 promoter (Atp8b1-S2 and Atp8b1-S3) in 293T cells but no changes in binding to the Atp8b1-S1 and Atp8b1-S4 regions (Fig. 6G). This result indicated that Bhlha15 can bind to the Atp8b1 promoter in CP, but only to the predicted sites in Atp8b1-S2 and Atp8b1-S3, not the predicted sites in Atp8b1-S1 and Atp8b1-S4, and the binding degree of Atp8b1-S2 is the highest. Then, to confirm the predicted binding site in Atp8b1-S2, we performed a luciferase assay, which indicated that Bhlha15 binds to the Atp8b1-S2 region and identified two binding sites in this region (Fig. 6H). In summary, we concluded that Bhlha15 regulates the transcriptional activity of Atp8b1 by binding to the Atp8b1 promoter.

## Discussion

**Fig. 7:**
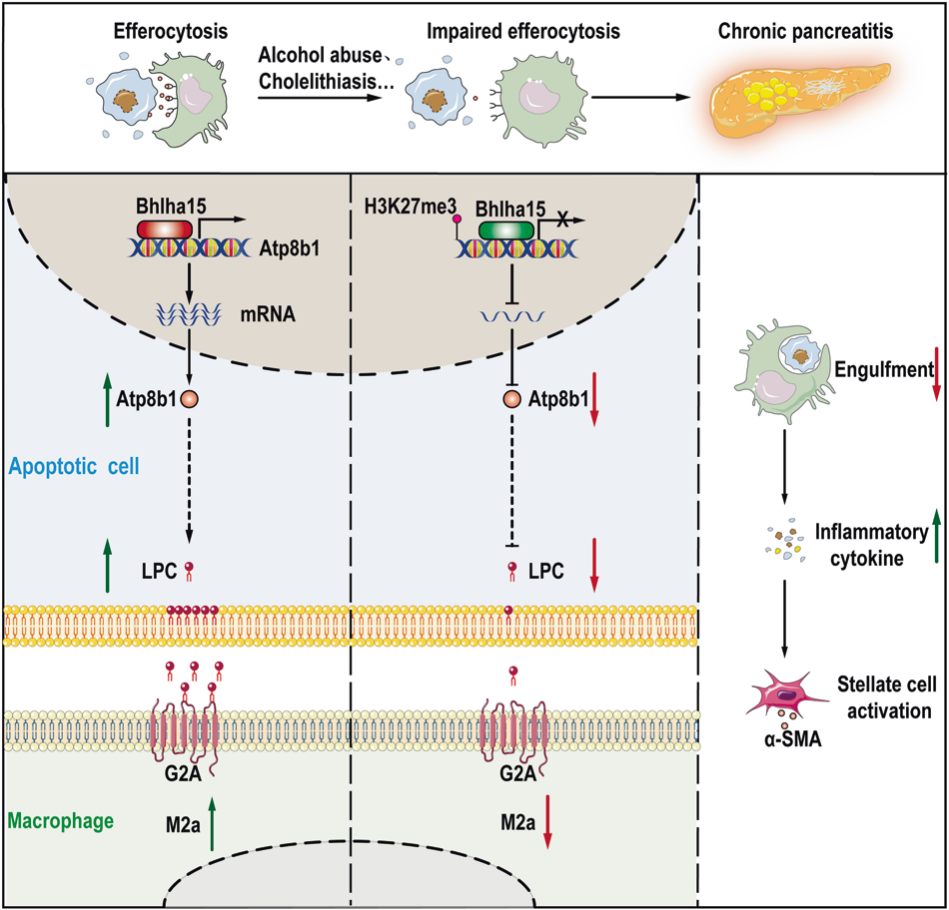
Graphical abstract. Mechanistic diagram showing how efferocytosis effect CP process through regulating Bhlha15/Atp8b1/LPC pathway to alter the regulation of phospholipid metabolism. Under normal conditions on the left, macrophages engulf apoptotic acinar cells through efferocytosis to resolve inflammation. Alcohol abuse and cholelithiasis can injury pancreas and impaire the efficiency of efferocytosis. Impaired efferocytosis decreases the efficiency of apoptotic cell clearance. H3K27me3 of histones suppresses Atp8b1 transcription, and decrease LPC concentration. This reduction means that less LPC is available to interact with G2A and fewer macrophages are attracted to engulf apoptotic cells, leading to further inflammation.

This study reveals increased inflammation and fibrosis caused by defective efferocytosis and a critical role for the acinar Bhlha15/Atp8b1/LPC pathway in the “find-me” signal of efferocytosis during CP. Bhlha15 was shown to bind to Atp8b1 promoter to increase its transcription, and Atp8b1 was found to increase the concentration of LPC and flip it to the outer surface of the cell membrane to induce macrophage-mediated clearance of apoptotic cells. Impaired efferocytosis decreased the efficiency of apoptotic cell clearance, which aggravated inflammation and fibrosis. H3K27me3 in the Atp8b1 promoter region suppressed transcription, which was followed by decreases in the LPC concentration. These changes in turn led to a decrease in macrophages that could engulf apoptotic cells and aggravated inflammation and fibrosis (Fig. 7). During CP development, the skewing of macrophage polarization from M1 to M2a resulted in defective efferocytosis. Experiments conducted to screen for transcription partners of Atp8b1 identified Bhlha15, an acinar-specific transcription factor, which was shown to induce Atp8b1 transcription and promote the Atp8b1/LPC pathway in acinar cells in CP. Commendamide, which binds to LPC receptor G2A, was shown to protect against acinar injury in CP. These results provide the first indication that the Bhlha15/Atp8b1/LPC pathway regulates efferocytosis in CP. However, much additional work is needed to translate these results into clinic.

Specific recognition of apoptotic cells is ensured by “find-me” signals, such as sphingosine-1-phosphate, LPC and CX3CL1. During apoptosis, LPC is found on the outer leaflet of the membrane and binds directly or indirectly, via bridging molecules (opsonins), to phagocytic receptors. In this study, proinflammatory M1 macrophage polarization was found to be increased and pro-efferocytic M2a polarization to be decreased in CP, with macrophage polarization and function found to depend on signals from apoptotic acinar cells. This defective efferocytosis aggravated inflammation and fibrosis during CP development, which lead to no effective treatment in clinic. We think that studies in *PRSS1*^*Tg*^ CP mice may potentially provide valuable mechanistic insights into defective efferocytosis in humans, and help to devise antifibrotic therapies t for controlling the progression of CP.

LPC, a phospholipid produced by the cleavage of phosphatidylcholine (PC) by phospholipase A2 (PLA2) [21], is an apoptosis-specific “find-me” signal that binds to the G-protein coupled receptor G2A on macrophages. Exposed LPC on the plasma membrane of dying cells can bind to IgM, which in turn binds to Fc receptors on phagocytes. Hence, LPC appears to function as both a “find-me” signal and an “eat-me” signal [9]. The regulation of inflammation by LPC plays different roles in inflammatory and infectious diseases [22]. In atherosclerosis, LPC exerts anti-inflammatory effects in vascular inflammation and atherosclerosis development because it can increase the expression of both extracellular superoxide dismutase [23]. During efferocytosis, LPC released by apoptotic cells can be recognized by G2A receptors in the plasma membrane of phagocytes and induce the migration and chemoattraction of phagocytes for efficient removal of apoptotic cells. In addition, phagocytes produce numerous anti-inflammatory and resolving factors to maintain homeostatic function and regulate tissue repair [24]. Therefore, the potential role of LPC in CP efferocytosis deserves deeper investigation and also provides a prime therapeutic strategy for CP patients.

The flippase Atp8b1 is a catalytic component of the P4-ATPase flippase complex, which is located in the plasma membrane and is important for the transport of phospholipids, particularly PCs, from the outer leaflet to the inner leaflet of the plasma membrane [25]. When Atp8b1 is not available to maintain the normal distribution of lipids between the two lipid layers in the plasma membrane, the canalicular membrane becomes too fragile to maintain its homeostasis [25], which leads to a pathological condition called progressive familial intrahepatic cholestasis type 1 (PFIC-1) [26]. Without intervention, patients with PFIC-1 generally develop complications of end-stage liver disease, such as cirrhosis and hepatic failure [27]. Furthermore, Atp8b1 deficiency is associated with pancreatitis [16], secretory diarrhoea, growth retardation, hearing impairment [28], hypothyroidism [29], and pneumonia [30]. Homeostasis of membrane phospholipids also plays a pivotal role in cellular oncogenesis and cancer progression which emphasize that Atp8b1 may be a new therapeutic target in cancer, but the etiology and pathogenesis still need to be characterized. In *PRSS1*^*Tg*^ CP model, we discovered that downregulation of Atp8b1 in mice treated with caerulein led to a decrease in the LPC concentration, but the exact mechanism that cause Atp8b1 downregulation needs deeper investigation in next work.

DNA methylation is an epigenetic mechanism in which a methyl group is transferred from *S*-adenosylmethionine (SAM) to the fifth carbon of a cytosine residue by a family of DNA methyltransferases (Dnmts) to form 5-methylcytosine (5mC). This process mediates gene repression by recruiting proteins involved in gene repression or inhibiting the binding of transcription factors to DNA [31]. Alterations in DNA methylation have been associated with cancer [32], and several other diseases and are of the utmost importance in mammalian embryonic development [33].

Histones are the central component of the nucleosomal subunit, forming an octamer containing the four core histone proteins (H3, H4, H2A, and H2B) [33, 34]. On histones, methylation can occur on arginine or lysine residues, and each lysine residue can be mono-, di-, or trimethylated. Histone methyltransferases (HMTs) are histone-modifying proteins catalyzing the addition of methyl groups to arginine and lysine residues. Methylation is linked to either gene activation or gene repression; H3K4, H3K36, and H3K79 methylation is associated with gene activation, while H3K9, H3K27, and H4K20 methylation indicates gene silencing [35]. H3K27me3 is also a repressive chromatin mark. The lysine methyltransferase (KMT) EZH2 (KMT6), an H3K27me2/3-specific KMT, is recruited to DNA damage sites, where it catalyses the addition of these histone methylation marks [36, 37], which may indicate that H3K27me3 participates in promoting DNA damage-associated transcriptional silencing.

Bhlha15, also called muscle intestine and stomach expression 1 (MIST1), is a basic helix-loop-helix transcription factor that is primarily expressed in the pancreas, stomach, intestine, colon. It is important for proper development of the exocrine pancreas, with its pancreatic expression restricted to acinar cells [38, 39]. Blockade of Bhlha15 in the final steps of differentiation was found to lead to a damaged exocrine pancreas with a defective exocytic ability [40]. Mice lacking the transcription factor Bhlha15 exhibited an altered stress response, increased sensitivity to caerulein-induced pancreatitis [41], altered cell proliferation [39]. After the induction of pancreatitis, serum amylase levels, necrosis, and tissue damage were elevated in MIST1 KO mice, suggesting that MIST1 plays a protective role in the development of pancreatitis [42]. Based on the above findings, it is clear that MIST1 plays a vital role in pancreatitis development. By searching Cistrome DB, we found that Atp8b1 is a putative target gene of Bhlha15. ChIP-qPCR and luciferase assays were performed to verify the prediction that two potential binding sites for Bhlha15 were located in the Atp8b1 promoter. These findings represent the discovery of a novel regulatory pathway for Atp8b1, which may provide therapy targets in Atp8b1-defiencent diseases, such as PFIC-1.

In nonalcoholic fatty liver disease (NAFLD), damaged hepatocytes and apoptotic cells trigger macrophage recruitment, which is followed by macrophage-mediated hepatic stellate cell (HSC) activation, leading to liver fibrosis [43]. In this process, apoptotic cells recruit macrophages by releasing soluble “find-me” signals, such as ATP, CX3CL1, and sphingosine-1-phosphate. The engulfment of apoptotic cells induced by an “eat-me” signal is triggered mainly by phosphatidylserine on the outer leaflet of apoptotic cells, which directly binds to the corresponding surface receptor on macrophages [44]. CP is a progressive and irreversible inflammatory and fibrotic disease with no cure. A study showed that alternatively activated macrophages (AAMs) are dominant in both mouse and human CP. In sharp contrast to AP, in which M1 macrophages predominate, CP favors AAMs (i.e., M2 macrophages) [45]. In addition, recent studies have indicated that macrophages function as regulators of fibrosis and that different macrophage subtypes play different roles in the initiation, maintenance and resolution of fibrosis [46, 47]. Correspondingly, in this study, the macrophage population was increased in CP tissues from mice compared to normal pancreas tissues. The proliferation of both resident macrophages and recruited monocytes that differentiated into macrophages partially contributed to the macrophage accumulation in CP [48].

Taken together, these findings provide important new insights into the defective efferocytosis in CP. Therapeutic targeting of Bhlha15/Atp8b1/LPC pathway or modulation of phagocytosis may serve as a promising approach to treat CP. Meanwhile, future studies may suggest new therapeutic opportunities for CP driven by excessive apoptosis, defective efferocytosis and impaired resolution and repair. Although we found a correlation between Atp8b1 and LPC, the details of their interaction need further clarification.

## Materials and methods

### Animal model

As previously reported [7], the *PRSS1*^*Tg*^ mice, weighed 20–25 g and aged 6–8 weeks, were used in this study for CP model by injecting caerulein (15 μg/ml) peritoneally at a dose of 50 μg/kg/h twice per week (one injection per hour for 8 hours) for four consecutive weeks. Control animals received normal saline. All animal experimental procedures were approved by the Institutional Animal Care and Use Committee of Southern Medical University.

### Atp8b1 and Bhlha15-overexpressing adeno-associated virus (AAV)

Adeno-associated virus (AAV) carrying full length of Atp8b1 and Bhlha15 genes were built to infect *PRSS1*^*Tg*^ mice respectively for genes overexpression to investigate their influence on apoptosis and inflammation during induction of CP. *PRSS1*^*Tg*^ mice harbouring adRNA was regarded as negative control (NC). VectorBuilder Inc (Guangzhou, China) provided all AAV vectors. The adenoviruses used in this study were listed in Supplemental methods.

### Luciferase assay

A series of mutants of Atp8b1 promoter (chr18:64660463-64661642) regions were respectively generated into promoter luciferase constructs, which were then inserted into the pGL3 vector. Subsequently, Bhlha15 sequence was cloned into pcDNA3.1 plasmid. 293T cells were grown in 24-well plates and co-transfected with plasmids containing a series of mutants of Atp8b1 promoter and Bhlha15 overexpression plasmids using the Lipofectamine 2000 Reagent (Invitrogen). Cellular extracts were measured for luciferase activity using the luciferase reporter assay system at 48 h. Each experiment was repeated three times.

### LC-MS/MS analysis

Phospholipid metabolites were extracted from pancreatic tissue samples according to a previously described protocol [49], following by analyzing via a 1290 Infinity II LC System (Agilent Technologies, USA) with a Waters Acquity UPLC BEH C18 column (2.1 × 150 mm; 1.7 μm particle size). Mass detection was performed in multiple reaction monitoring (MRM) mode with an Agilent 6495 triple quadrupole system equipped with Agilent Jet Stream thermal gradient focusing technology, which uses an enhanced ESI technique to confine the nebulizer spray.

### Immunohistochemistry, Immunofluorescence assay, and Masson’s trichrome staining

H&E staining analysis and immunohistochemistry of pancreatic tissues slides for F4/80, CD86, TLR2, CD206, and ARG1 were carried out according to protocol displayed before [50]. Immunofluorescence assay was performed to visualize α-SMA and F4/80 expression. Masson’s trichrome staining was to evaluate α-SMA and Collagen content in pancreatic tissue by Masson’s trichrome pancreatic kit according to manufacturer’s specifications. Please see the Supplemental Methods for more details.

### Western blotting and qRT-PCR

Total protein was extracted from the mouse pancreatic tissues using the Tissue or Cell Total Protein Extraction Kit (C510003; Sangon, Shanghai, China) following the manufacturer’s protocol. Antibodes used in this study includes anti-Atp8b1 and anti-Bhlha15. The total mRNA expression of Atp8b1 in pancreatic tissue was quantification by Quantitative RT-PCR. More details exsited in Supplemental Methods.

### Enzyme-linked immunosorbent Assay (ELISA)

Supernatants from mice pancreatic tissues were collected and stored at -80 °C after removal of serum by centrifugation. The protein concentration of IL-1β, IL-6, and TNF-α in the pancreatic tissues were detected using specific ELISA kits (Signalway Antibody) according to the manufacturer’s instructions.

### Transferase-mediated d-UTP nick-end-labelling (TUNEL) assay

Tissue slides infiltrated in Paraffin were de-waxed with dimethylbenzene and hydrated with ethanol concentration gradient. TUNEL assay was used to detect DNA fragmentation in apoptotic cells with ApopTag peroxidase Kit (MP Biomedicals, Illkirch, France) as declared by the specification. The green staining in cell nucleus represented DNA cleavage and fragmentation in apoptosis cell and DAPI staining was bind to cell nucleus to localize apoptosis cell. More than three biological replicates were performed.

### Depletion of macrophages

Selective macrophage depletion was achieved with intraperitoneal injection of Clodronate Liposomes (CLOs). This method of macrophage depletion was designed based on its unique ability of phagocytic activity. The *PRSS1*^*Tg*^ mice were injected with CLOs (FormuMax, F70101C-N-2) through the intraperitoneal injection for the depletion of macrophages. CLOs was administered at a dose of 200 μl 24 h prior to caerulein injection and again 24 h after each caerulein injection to deplete resident and infiltrating macrophages twice weekly during CP model induction.

### ATAC‑seq

Pancreatic tissues from *PRSS1*^*Tg*^ mice were micro-dissected under the microscope into pancreatic acinar cells. After dissociation, cells were resuspended in PBS. Following the ATAC-seq protocol, approximately 50,000 cells were used for cell lysis and transposase (2.5 μl transposase in 50 μl buffer) treatment at 37 °C for 30 min. The cell numbers for the experiments were carefully optimized through several trials to achieve clear nucleosome patterns and ensure the qualities of the libraries. The DNA fragments were then purified using MinElute PCR Purification Kit (Qiagen, Cat No. 28004) and amplified by PCR. Both quality and quantity of ATAC-seq libraries were examined by Bioanalyzer. The ATAC-seq libraries were sent for 75 bp paired-end sequencing on Illumina NextSeq 500. Please see the Supplemental Methods for more details.

### Chromatin immunoprecipitation with sequencing (ChIP‑seq)

Cells (1 × 10^7^) were cross-linked in 1% formaldehyde for 20 min, quenched with 125 mM glycine for 5 min. After lysing in 0.5% Igepal CA-630 in PBS supplemented with protease inhibitors for 10 min, nuclei were pelleted and digested with 1 μl micrococcal nuclease for 3 min at 37 °C to fragment chromatin. Next, the fragment chromatin was resuspended in SDS lysis buffer (pH 8.0, 1% SDS, 150 mM NaCl, and 5 mM EDTA) and briefly sonicated. The reads were aligned to the mouse genome (mm10) using Bowtie2 (version 2.3.3.1) with default parameters. Duplicated reads were removed using SAM tools (v 1.2) and deep Tools2 (v 2.5.2) was further used to subtract input signal and normalize the reads to the same sequencing depth. P300 ChIP-seq peaks at E11.5 forebrain were downloaded from the supplementary files in the paper. All peak files were converted from mm9 to mm10 using Lift Over tool from UCSC genome browser when necessary.

### Flow cytometry

The preparation of single-cell suspension of pancreatic tissue was executed by a collagenase digestion method described as before for flow cytometry analysis [51]. Pancreatic tissue was cut into small pieces(0.5 cm^3^) and digested in fluorescence‐activated cell sorting (FACS) Buffer (HBSS + 10% FCS) containing 2 mg/ml collagenase type IV. The tissues were incubated in a shaker at 37 °C for 15 min and vortexed at a low speed for 20 s. After digestion, single cells were passed through a 70 μm cell strainer. To distinguish various macrophages, we added anti-F4/80-PE/Cy7 (TONBO Biosciences), CD86-APC (TONBO Biosciences), and CD206-PE antibodies (Invitrogen) for surface staining and incubated for 30 min in the dark at 4 °C. The cells were stained with an isotype-matched control antibody as a negative control. Cells were washed with FACS buffer and sorted by FACSusing NovoCyte Flow Cytometer (ACEA Biosciences, Inc., San Diego, CA, USA). Data obtained were acquired at the same time and analyzed using FlowJo software.

### Statistical analysis

GraphPad Prism 8.0 and SPSS 24.0 software were used for statistical analysis. The continuous variables are expressed as mean ± standard error. Significant differences between two groups were analyzed by Student’s t test, and one-way analysis of variation was performed to investigate the differences among more than two groups. Significant differences were defined by a *P* value of < 0.05.

### Access to data

Raw data of ChIP-seq and RNA-sequence have been submitted to the NCBI Sequence Read Archive under Accession number PRJNA777968. More data related to this paper may be acquired from the authors.

## Supplementary information

A reproducibility checklist

Author Contribution Statement

Supplementary Figure 1

Supplementary Figure 2

Supplementary Figure 3

Supplementary Figure 4

Supplementary Information

Supplementary Methods

Table S1

Western blot
