# Correction: Acinar ATP8b1/LPC pathway promotes macrophage efferocytosis and clearance of inflammation during chronic pancreatitis development

**DOI:** 10.1038/s41419-022-05374-8

**Published:** 2022-11-07

**Authors:** Wan-jun Yang, Rong-chang Cao, Wang Xiao, Xiao-lou Zhang, Hao Xu, Meng Wang, Zhi-tao Zhou, Huo-ji Chen, Jia Xu, Xue-mei Chen, Jun-ling Zeng, Shu-ji Li, Min Luo, Yan-jiang Han, Xiao-bing Yang, Guo-dong Feng, Yu-heng Lu, Yuan-yuan Ni, Chan-gui Wu, Jun-jie Bai, Zi-qi Yuan, Jin Jin, Guo-wei Zhang

**Affiliations:** 1 Division of Hepatobiliopancreatic Surgery, Department of General Surgery, Nanfang Hospital, Southern Medical University, Guangzhou, China; 2 Nanfang PET Center, Nanfang Hospital, Southern Medical University, Guangzhou, China; 3 Department of the Electronic Microscope Room, Central Laboratory, Southern Medical University, Guangzhou, China; 4 School of Traditional Chinese Medicine, Southern Medical University, Guangzhou, China; 5 Department of Pathophysiology, Southern Medical University, Guangzhou, China; 6 Department of Occupational Health and Medicine, Guangdong Provincial Key Laboratory of Tropical Disease Research, School of Public Health, Southern Medical University, Guangzhou, China; 7 Laboratory Animal Research Center of Nanfang Hospital, Southern Medical University, Guangzhou, China; 8 Guangdong-Hong Kong-Macao Greater Bay Area Center for Brain Science and Brain-Inspired Intelligence, Southern Medical University, Guangzhou, China; 9 Department of Laboratory Medicine, Nanfang Hospital, Southern Medical University, Guangzhou, China; 10 Department of Nuclear Medicine, Nanfang Hospital, Southern Medical University, Guangzhou, China; 11 Division of Nephrology, Nanfang Hospital, Southern Medical University, National Clinical Research Center for Kidney Disease, State Key Laboratory of Organ Failure Research, Guangdong Institute, Guangzhou, China; 12 Southern Medical University, Guangzhou, China; 13 Department of Gynaecology and Obstetrics, Nanfang Hospital, Southern Medical University, Guangzhou, China

**Keywords:** Infectious diseases, Inflammation

Correction to: *Cell Death and Disease* 10.1038/s41419-022-05322-6, published online 22 October 2022

The original version of this article contained and error in an author name. “Wang-jun Yang” is incorrect, and should be modified to “Wan-jun Yang”. The authors apologize for the error. The original article has been corrected.

