# Supplementary material for: Acinar ATP8b1/LPC pathway promotes macrophage efferocytosis and clearance of inflammation during chronic pancreatitis development
Source: Cell Death Dis. 2022 Oct 22;13(10):893. doi: 10.1038/s41419-022-05322-6 (PMC9588032; doi:10.1038/s41419-022-05322-6)
Supplement: Supplementary file 2 — Author Contribution Statement [file 41419_2022_5322_MOESM2_ESM.pdf]

DECLARATION OF CONTRIBUTIONS TO ARTICLE

ADMC

Manuscript Number: CDDIS-22-2096RR

Journal Name: *Cell Death & Disease* (the 'Journal')

Proposed Title of the Contribution:

Acinar ATP8b1/LPC pathway promotes macrophage efferocytosis and clearance of inflammation during chronic pancreatitis development (the 'Contribution)

Author(s):

Wang-jun Yang, Rong-chang Cao, Wang Xiao, Xiao-lou Zhang, Hao Xu, Meng Wang, Zhi-tao Zhou, Huo-ji Chen, Jia Xu, Xue-mei Chen, Jun-ling Zeng, Shu-ji Li, Min Luo, Yan-jiang Han, Xiao-bing Yang, Guo-dong Feng, Yu-heng Lu, Yuan-yuan Ni, Chan-gui Wu, Jun-jie Bai, Zi-qi Yuan,Jin Jin,Guo-wei Zhang (the 'Authors')

For all *CDD* articles, each person named as an author in the published version must be able to show he or she has contributed substantially to the article.

Authorship credit should be based on 1) substantial contributions to conception and design, acquisition of data, or analysis and interpretation of data; 2) drafting the article or revising it critically for important intellectual content; and 3) final approval of the version to be published. Authors should meet conditions 1, 2 and 3.

Any person who cannot be shown to have made a substantial contribution to the article cannot be listed as an author in the final version. The name of any person who is deemed to have made a minor contribution can, however, appear in the Acknowledgments section of the article.

Please complete the table below to indicate the contributions of all named authors to the manuscript.

Author Full Name:	Specification of Contribution to the Manuscript:
Wang-jun Yang	Performed the experiments, analyzed the data, and drafted the manuscript.
Rong-chang Cao	Performed the experiments, analyzed the data, and drafted the manuscript.
Guo-wei Zhang	Performed study concept and design. Performed the research. Analyzed the data. Wrote and revised the paper.
Jin Jin	Performed study concept and design.Performed the research.
Wang Xiao	Performed the research. Analyzed the data.
Hao Xu,Xiao-lou Zhang	Establish chronic pancreatitis mice model.Analyzed the data.
Meng Wang	Provided technical and material support.
Zhi-tao Zhou,Huo-ji Chen	Provided technical and material support.Data collection and analysis.
Jia Xu,Zi-qi Yuan	Performed the research.Data collection.
Jun-ling Zeng,Shu-ji Li,Min Luo	Provided technical and material support.
Yan-jiang Han	Provided technical and material support and analyzed the data.
Guo-dong Feng,Yu-heng Lu	collected date and performed animal experiment.
Chan-gui Wu,Jun-jie Bai	Data visualization and creation of animal models.
Xiao-bing Yang,Yuan-yuan Ni	Data visualization and creation of animal models.
Xue-mei Chen	Provided technical and material support.Data collection and analysis.

Please complete the table below to indicate the contributions of all named authors to the figures.

Figure 1:

In this figure, Wang-jun Yang, Rong-chang Cao and Wang Xiao performed experiments and analyzed the data.Zhi-tao Zhou,Huo-ji Chen provided technical and material support.Guo-wei Zhang revised this figure.Wang-jun Yang assembled this figure.

Figure 2:

In this figure,Wang-jun Yang,Jin Jin and Zi-qi Yuan performed the experiments and analyzed the data.Meng Wang Provided technical support.Xiao-bing Yang and Zhi-tao Zhou collected the data and visualized the data.Wang-jun Yang and Rong-chang Cao assembled this figure.Jin Jin revised this figure.

Figure 3:

In this figure,Hao Xu,Xiao-lou Zhang and Jia Xu,Zi-qi Yuan establish chronic pancreatitis mice model, performed experiments and collected the data.Xiao-bing Yang,Yuan-yuan Ni were responsible for designing the layout and assembling this figure.

Figure 4:

In this figure,Wang-jun Yang, Rong-chang Cao and Xue-mei Chen performed the experiments.Zhi-tao Zhou,Huo-ji Chen and Wang Xiao analyzed the data.Wang-jun Yang, Rong-chang Cao were responsibe for assembling this figure.

Figure 5:

In this figure,Guo-dong Feng,Yu-heng Lu and Rong-chang Cao performed the experiments,Jun-ling Zeng,Shu-ji Li and Min Luo provided technical and material support.Yan-jiang Han and Xue-mei Chen analyzed the data.Rong-chang Cao were responsibe for assembling this figure.

Figure 6、7:

In this figure,Jia Xu,Zi-qi Yuan and Jin Jin performed the experiments,Guo-wei Zhang and Yan-jiang Han analyzed the data.Chan-gui Wu,Jun-jie Bai and Wan-jun Yang prepared all panel and assembled the figure.

Signed for and on behalf of the Author(s):	Print Name:	Date:
Wan-jun Yang	Wan-jun Yang	2022-09-23
