# Supplementary figures and images for: Acinar ATP8b1/LPC pathway promotes macrophage efferocytosis and clearance of inflammation during chronic pancreatitis development
Source: Cell Death Dis. 2022 Oct 22;13(10):893. doi: 10.1038/s41419-022-05322-6 (PMC9588032; doi:10.1038/s41419-022-05322-6)

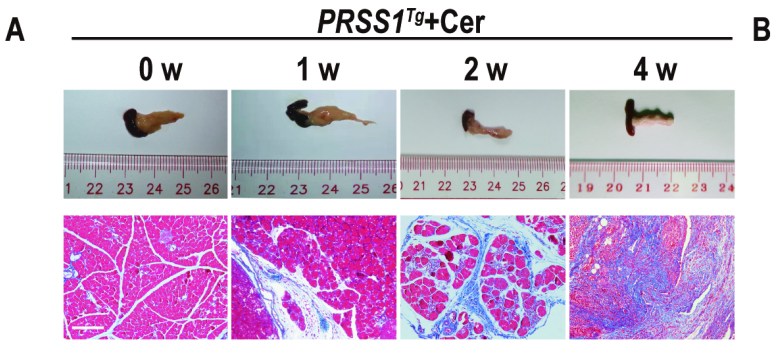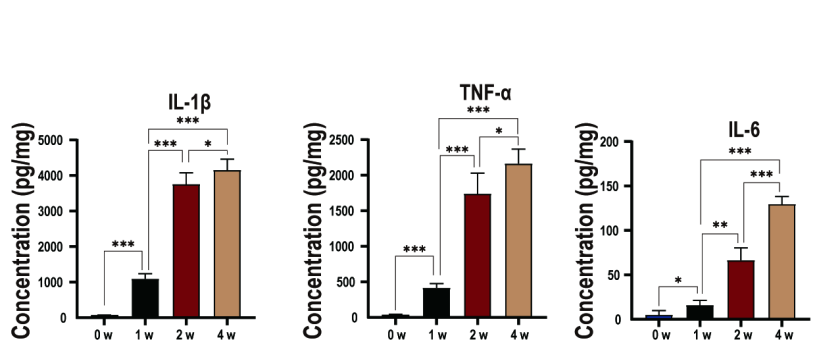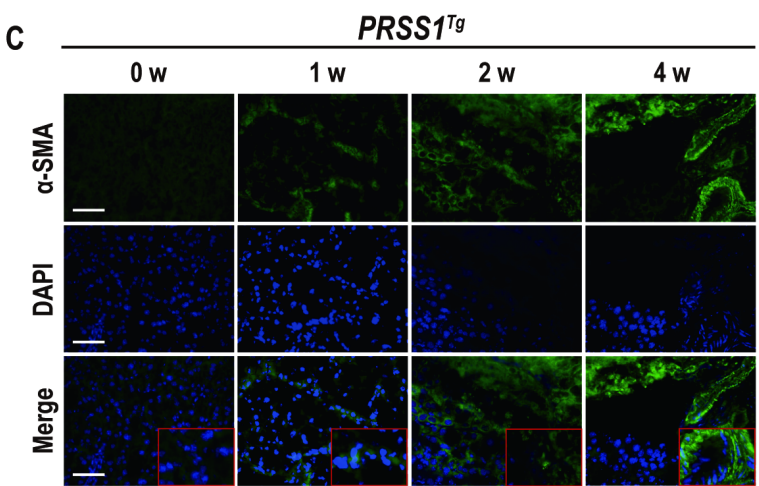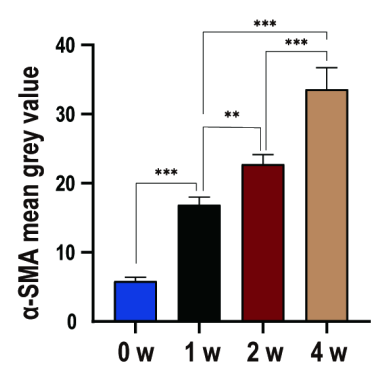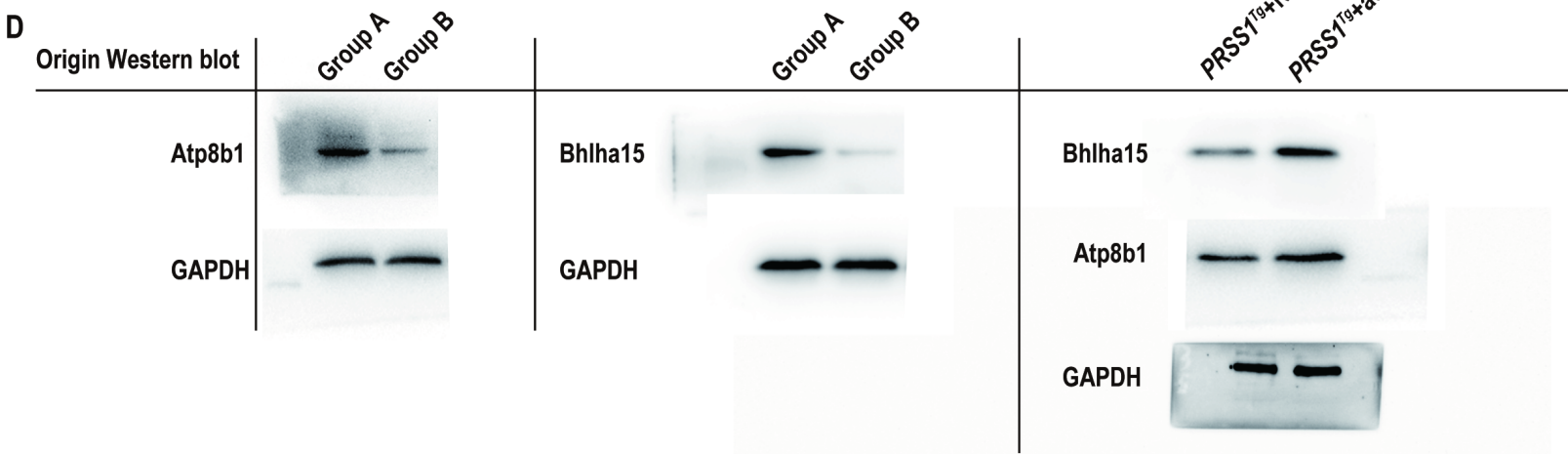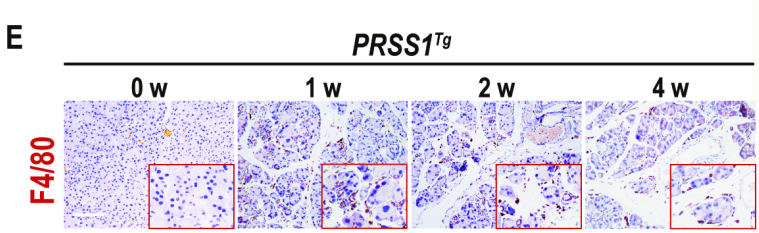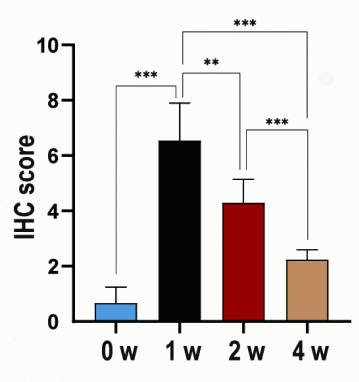

Supplement: Supplementary file 3 — Supplementary Figure 1 [file 41419_2022_5322_MOESM3_ESM.pdf]

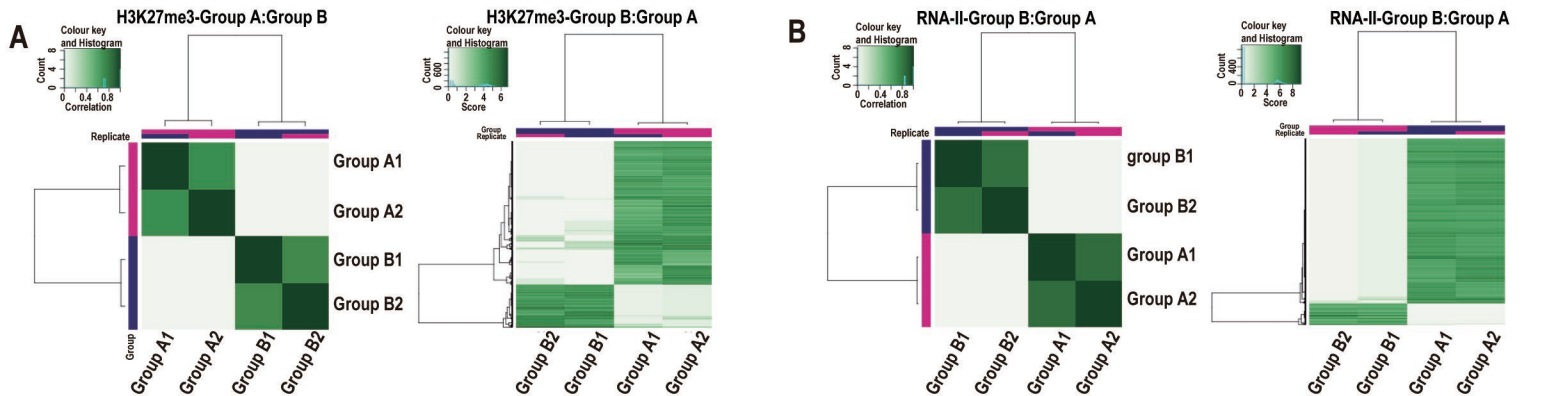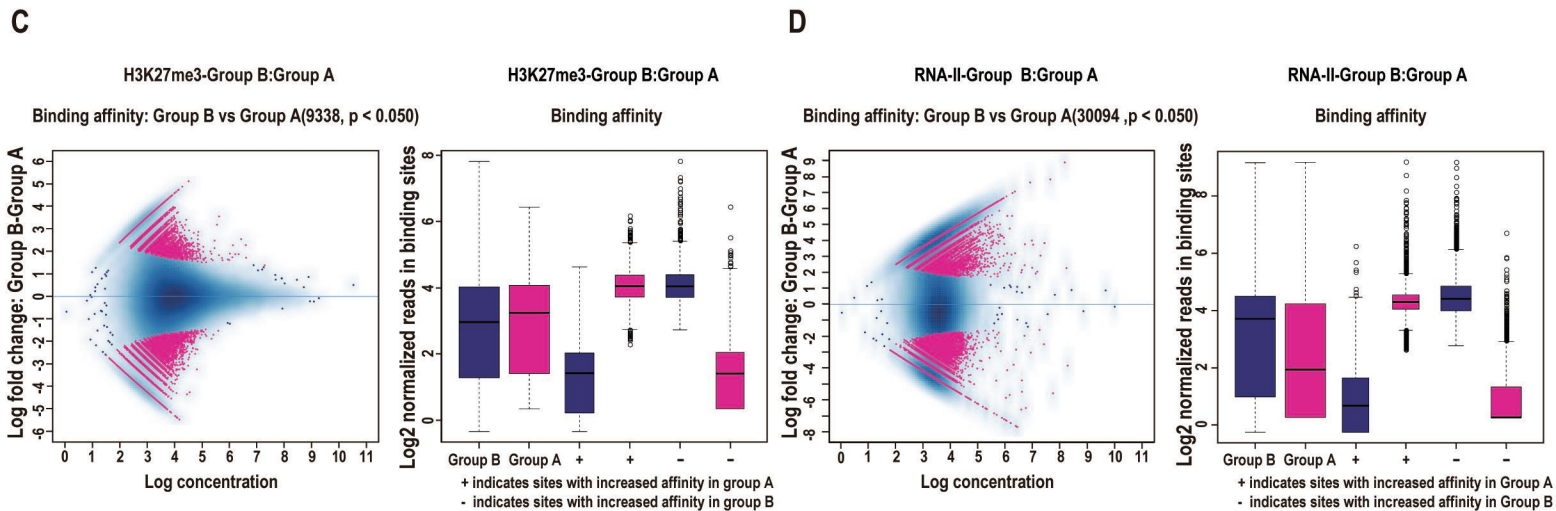

Supplement: Supplementary file 4 — Supplementary Figure 2 [file 41419_2022_5322_MOESM4_ESM.pdf]

A

# ssAAV.CAG.mAtp8b1-3FLAG.PA48

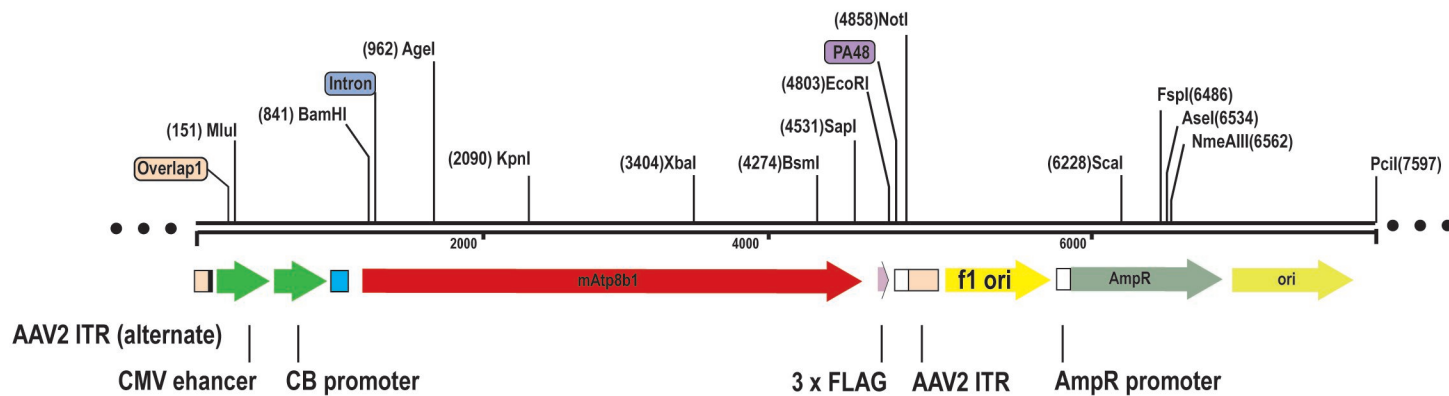

# ssAAV.CAG.EGFP.WPRE.SV40pA

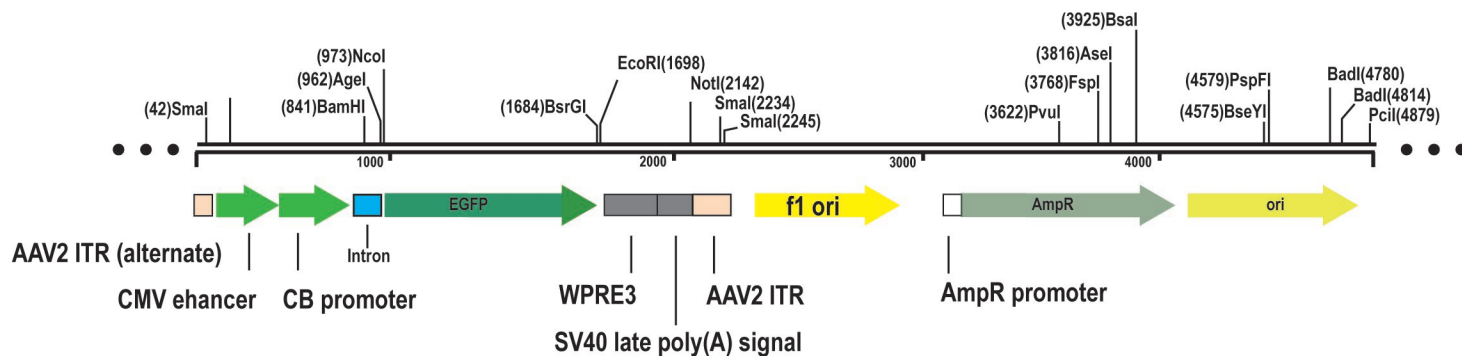

B

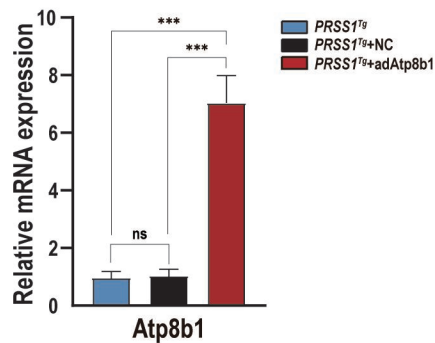

C

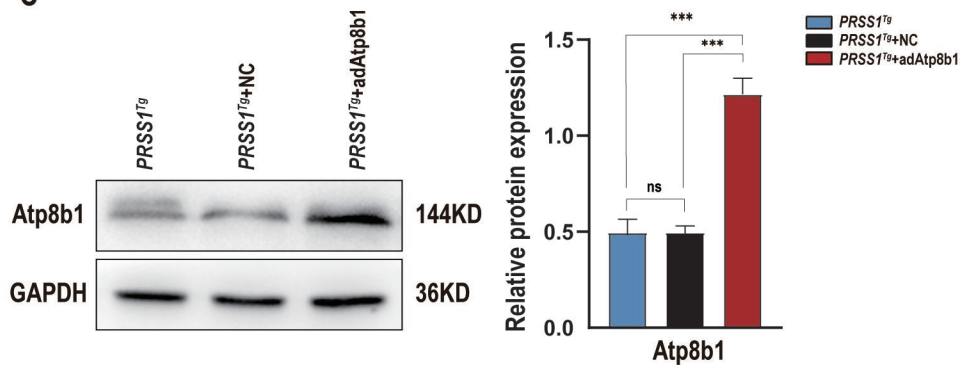

Supplement: Supplementary file 5 — Supplementary Figure 3 [file 41419_2022_5322_MOESM5_ESM.pdf]

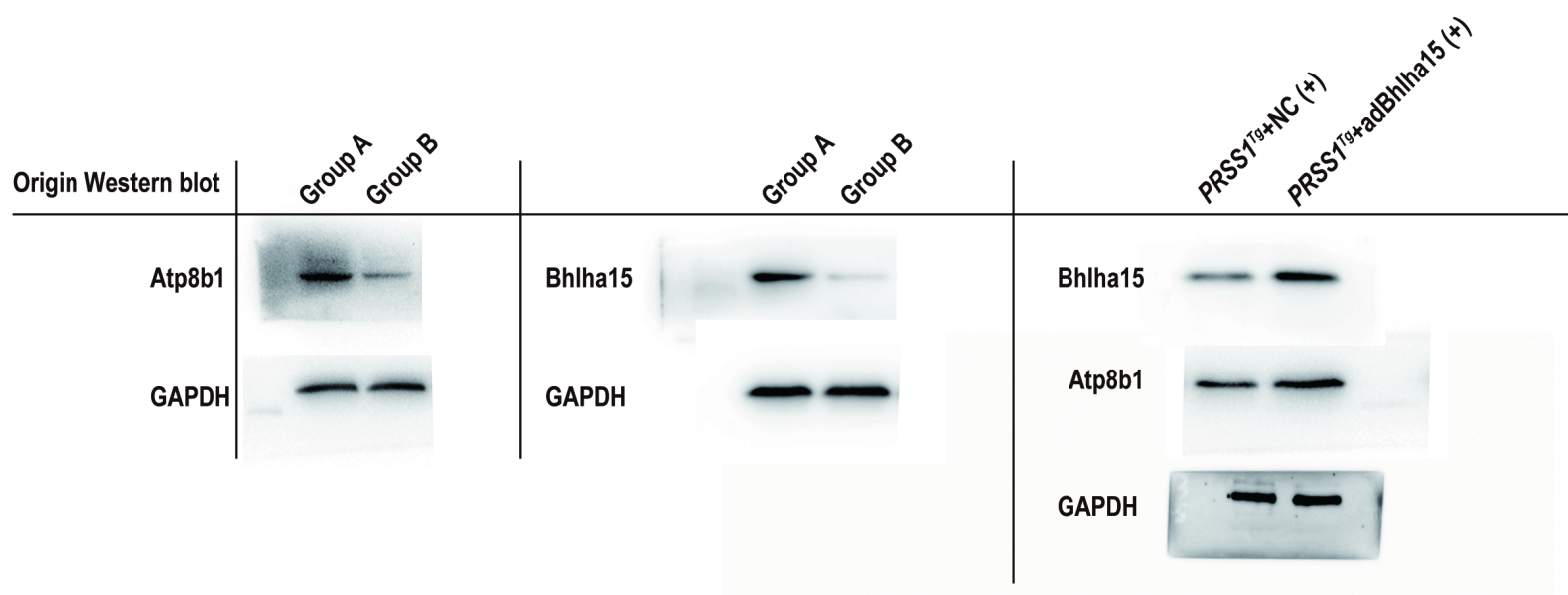

Supplement: Supplementary file 10 — Western blot [file 41419_2022_5322_MOESM10_ESM.pdf]
