# Supplementary material for: Acinar ATP8b1/LPC pathway promotes macrophage efferocytosis and clearance of inflammation during chronic pancreatitis development
Source: Cell Death Dis. 2022 Oct 22;13(10):893. doi: 10.1038/s41419-022-05322-6 (PMC9588032; doi:10.1038/s41419-022-05322-6)
Supplement: Supplementary file 6 — Supplementary Figure 4 [file 41419_2022_5322_MOESM6_ESM.pdf]

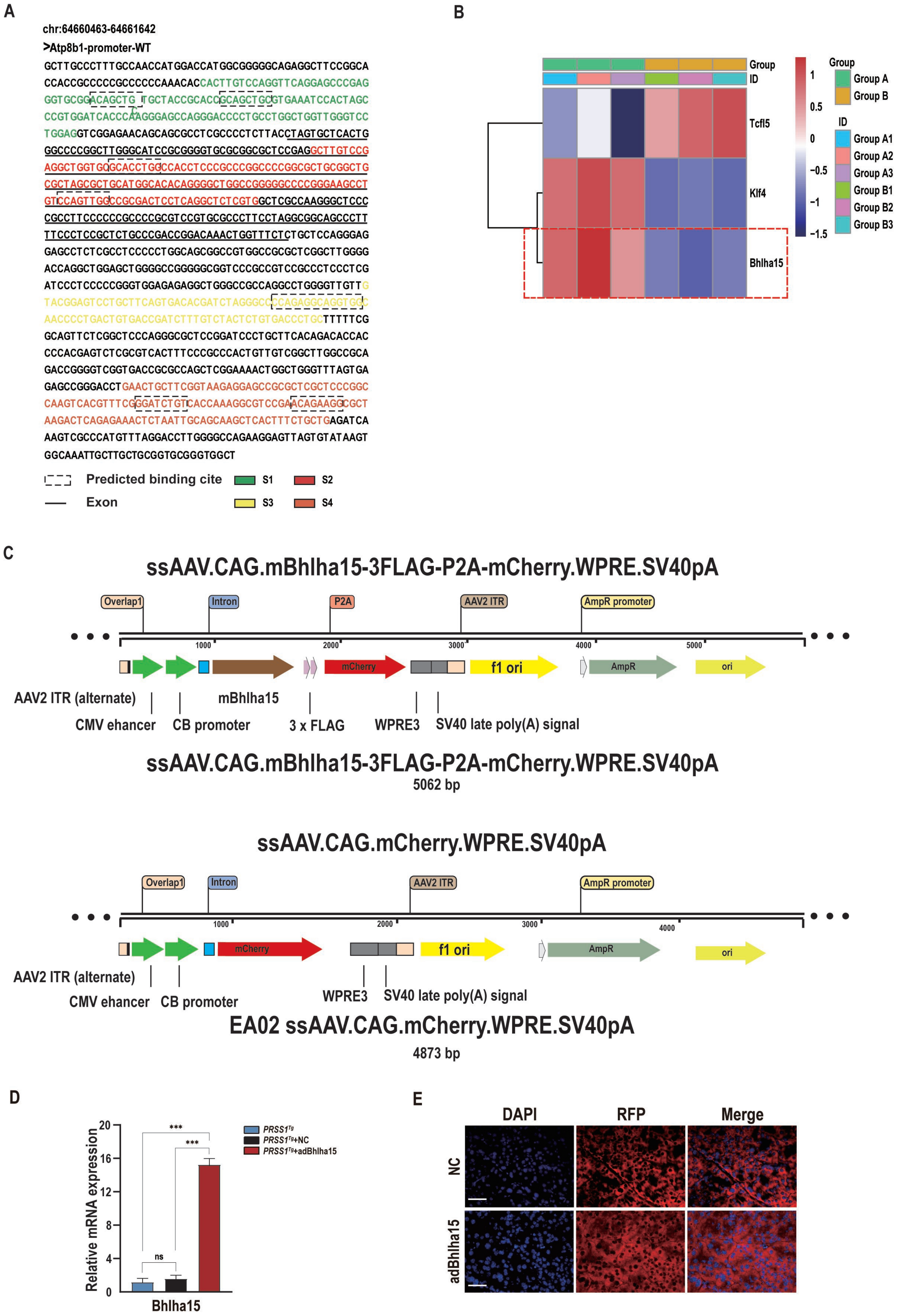

A
chr:64660463-64661642
>Atp8b1-promoter-WT
GCTTGCCCTTTGCCAACCATGGACCATGGCGGGGGCAGAGGCTTCCGGCA
CCACCGCCCCCGCCCCCCAAACACCACTTGTCCAGGTTCAGGAGCCCGAG
GGTGCGGACAGCTGTGCTACCGCACCGCAGCTGCGTGAAATCCACTAGC
CCGTGGATCACCCAAGGGAGCCAGGGACCCCTGCCTGGCTGGTTGGGTCC
TGGAGGTCGGAGAACAGCAGCGCCTCGCCCCTCTTACCTAGTGCTCACTG
GGCCCCGGCTTGGGCATCCGCGGGGTGCGCGGCGCTCCGAGGCTTGTCCG
AGGCTGGTGCGCACCTGGCCACCTCCCGCCCGGCCCCGGCGCTGCGGCTG
CGCTAGCGCTGCATGGCACACAGGGGCTGGCCGGGGGCCCCGGGAAGCCT
GTCCAGTTGGCCGCGACTCCTCAGGCTCTCGTGGCTCGCCAAGGGCTCCC
CGCCTTCCCCCCGCCCCGCGTCCGTGCGCCCTTCCTAGGCGGCAGCCCTT
TTCCCTCCGCTCTGCCCGACCGGACAAACTGGTTTCTCTGCTCCAGGGAG
GAGCCTCTCGCCTCCCCCTGGCAGCGGCCGTGGCCGCGCTCGGCTTGGGG
ACCAGGCTGGAGCTGGGGCCGGGGGCGGTCCCGCCGTCCGCCCTCCCTCG
ATCCCTCCCCCGGGTGGAGAGAGGCTGGGCCGCCAGGCCTGGGGTTGTTG
TACGGAGTCCTGCTTCAGTGACACGATCTAGGGCCCCAGAGGCAGGTGGC
AACCCCTGACTGTGACCGATCTTTGTCTACTCTGTGACCCTGCTTTTTCG
GCAGTTCTCGGCTCCCAGGGCGCTCCGGATCCCTGCTTCACAGACACCAC
CCCACGAGTCTCGCGTCACTTTCCCGCCCACTGTTGTCGGCTTGGCCGCA
GACCGGGGTCGGTGACCGCGCCAGCTCGGAAAACTGGCTGGGTTTAGTGA
GAGCCGGGACCTGAACTGCTTCGGTAAGAGGAGCCGCGCTCGCTCCCGGC
CAAGTCACGTTTCGGGATCTGTCACCAAAGGCGTCCGAACAGAAGGCGCT
AAGACTCAGAGAAACTCTAATTGCAGCAAGCTCACTTTCTGCTGAGATCA
AAGTCGCCCATGTTTAGGACCTTGGGGCCAGAAGGAGTTAGTGTATAAGT
GGCAAATTGCTTGCTGCGGTGCGGGTGGCT
Predicted binding cite
Exon
S1
S2
S3
S4
B
Group
ID
Tcfl5
Klf4
Bhlha15
1
0.5
0
−0.5
−1
−1.5
Group
Group A
Group B
ID
Group A1
Group A2
Group A3
Group B1
Group B2
Group B3
C
ssAAV.CAG.mBhlha15-3FLAG-P2A-mCherry.WPRE.SV40pA
Overlap1
Intron
P2A
AAV2 ITR
AmpR promoter
1000
2000
3000
4000
5000
mCherry
f1 ori
AmpR
ori
AAV2 ITR (alternate)
mBhlha15
CMV ehancer
CB promoter
3 x FLAG
WPRE3
SV40 late poly(A) signal
ssAAV.CAG.mBhlha15-3FLAG-P2A-mCherry.WPRE.SV40pA
5062 bp
ssAAV.CAG.mCherry.WPRE.SV40pA
Overlap1
Intron
AAV2 ITR
AmpR promoter
1000
2000
3000
4000
mCherry
f1 ori
AmpR
ori
AAV2 ITR (alternate)
CMV ehancer
CB promoter
WPRE3
SV40 late poly(A) signal
EA02 ssAAV.CAG.mCherry.WPRE.SV40pA
4873 bp
D
Relative mRNA expression
20
16
12
8
4
0
***
***
ns
PRSS1Tg
PRSS1Tg+NC
PRSS1Tg+adBhlha15
Bhlha15
E
DAPI
RFP
Merge
NC
adBhlha15
