# Supplementary material for: Acinar ATP8b1/LPC pathway promotes macrophage efferocytosis and clearance of inflammation during chronic pancreatitis development
Source: Cell Death Dis. 2022 Oct 22;13(10):893. doi: 10.1038/s41419-022-05322-6 (PMC9588032; doi:10.1038/s41419-022-05322-6)
Supplement: Supplementary file 7 — Supplementary Information [file 41419_2022_5322_MOESM7_ESM.docx]

**Figure S1. Aggravation of the phenotype with extension of the induction time in the CP mouse model.**

**(A)** Pancreatic morphology and fibrosis were evaluated by Masson’s trichrome staining of pancreatic tissues from caerulein-treated *PRSS1^T^*^g^ mice at weeks 0, 1, 2 and 4. (**B**) Quantitative analysis of IL-1β, IL-6, and TNF-α levels in pancreatic tissues from caerulein-treated *PRSS1^T^*^g^ mice. (**C)** The expression of α-SMA in pancreatic tissues from caerulein-treated *PRSS1^Tg^* mice at weeks 0, 1, 2 and 4 was detected by immunofluorescence staining, and the mean grey values calculated by ImageJ were used to quantitatively analyse this expression. The mean grey values was calculated using integrated density divided by area. (**D**) Western blot analysis of Atp8b1 and Bhlha15 in pancreatic tissue from mice in Group A and B was performed in triplicate. **(E)** The expression of F4/80 in the pancreatic tissues from caerulein-treated *PRSS1^Tg^* mice at weeks 0, 1, 2 and 4 was detected by immunohistochemical staining. The positive staining sections and staining intensity were reviewed and scored by two experienced pathologists with double blind manner. The final score was defined as staining number score multiplied by staining color score. The data are presented as the means ± SDs. ns, no significant difference; * p ≤ 0.05; ** p ≤ 0.01; *** p ≤ 0.001.

**Figure S2. Role of Atp8b1 in the *PRSS1^Tg^* CP mouse model.**

**(A)** The distribution of H3K27me3 enrichment was analysed with the DiffBind algorithm, which was used to calculate the spatial correlations between the genomic distributions of histone marks in Group A and B. The heatmaps of the DiffBind analysis results show the differential peaks for H3K27me3 in Group A and B. **(B)** DiffBind analysis was performed to reveal spatial correlations between RNA pol II occupancy in Group A and Group B by analysing the differential peaks of RNA pol II in Group A and Group B, and the results are shown in heatmaps. **(C)** MA plot for differential analysis of H3K27me3 ChIP-seq peaks in Group A and Group B using DiffBind. The effectiveness of distinguishing differential peaks is shown. **(D)** MA plot for differential analysis of RNA pol II ChIP-seq peaks in Group A and Group B using DiffBind. The effectiveness of distinguishing differential peaks is shown.

**Figure S3. Delivery of AdAtp8b1 to the pancreas in *PRSS1^Tg^* mice for Atp8b1 overexpression.**

**(A)** Schematic diagrams of NC-Atp8b1 and adAtp8b1. To investigate the effect of Atp8b1 on efferocytosis and phospholipid metabolism, the pancreases of *PRSS1^Tg^* mice were infected with adenoviral vectors harboring full-length Atp8b1 for Atp8b1 overexpression or a scrambled adRNA for the negative control, respectively. The mRNA (**B**) and protein (**C**) expression levels of Atp8b1 in pancreatic tissue from *PRSS1^T^*^g^ mice in the negative control groups and Atp8b1-overexpressing were determined by quantitative RT–PCR and western blotting.

**Figure S4. Role of Bhlha15 in the *PRSS1^Tg^* CP mouse model.**

(**A**) Differential ATAC-seq peak sequences near the TSS of Atp8b1 and the sequences of predicted Bhlha15 binding sites in Group A and Group B. The dotted boxes indicate the predicted binding sites, and the underlined text indicates the exon. The S1, S2, S3 and S4 regions detected by ChIP-qPCR are shown in green, red, yellow and orange text, respectively (**B**). Heatmap showing the differential expression of 3 genes determined by prediction of transcription factor binding to the open chromatin regions near the TSS of Atp8b1 using Jaspar (threshold score, > 90). (**C**) Schematic diagrams of NC-Bhlha15 and adBhlha15. To investigate the effect of Bhlha15 on efferocytosis and phospholipid metabolism, the pancreases of *PRSS1^Tg^* mice were infected with adenoviral vectors harboring full-length Bhlha15 for Bhlha15 overexpression or a scrambled adRNA for the negative control, respectively.(D)The mRNA expression level of Bhlha15 in pancreatic tissue from *PRSS1^T^*^g^ mice in the negative control groups and Bhlha15-overexpressing were measured by quantitative RT–PCR.(E) The adenoviral transduction efficiency of NC-Bhlha15 and adBhlha15 was assessed by quantifying RFP-fluorescence intensity using fluorescence microscopy.
