# Supplementary material for: Acinar ATP8b1/LPC pathway promotes macrophage efferocytosis and clearance of inflammation during chronic pancreatitis development
Source: Cell Death Dis. 2022 Oct 22;13(10):893. doi: 10.1038/s41419-022-05322-6 (PMC9588032; doi:10.1038/s41419-022-05322-6)
Supplement: Supplementary file 8 — Supplementary Methods [file 41419_2022_5322_MOESM8_ESM.docx]

**Supplementary materials and methods**

**Atp8b1 and Bhlha15-overexpressing** **adeno-associated virus (AAV)**

In order to build Atp8b1 and Bhlha15 overexpression CP *PRSS1^Tg^* mice model to investigate the effect of Atp8b1 and Bhlha15 on apoptosis and inflammation, adenoviral vectors harboring full-length Atp8b1 and Bhlha15 were built and then injected into pancreas by intraductal administration respectively as reported before[1], a scrambled adRNA was designed as the negative control (NC). Virus suspension with a titer of 1.5×10^11^ at a dose of 100 μl each mouse (20-25g) was injected into the pancreatic tissues. Immunofluorescence and western-blotting were peformed to determine genes expression 2 weeks late after virus delivery. While virus injection was finished nine days late, *PRSS1^Tg^* mice were treated with caerulein intraperitoneally to establish CP model subsequently. All adenoviral vectors were purchased from VectorBuilder Inc. (Guangzhou, China).

**Immunofluorescence assay and Immunohistochemistry**

For visualization of F4/80, α-SMA expression in pancreatic tissue, immunofluorescence assay was performed using primary antibodies tageting F4/80 (Bioss; diluted 1: 100) and α-SMA (Abcam; diluted 1:100) respectively. Pancreatic tissues from mice were collected and sliced into 5 μm tissue sections, and then fixed with 4% paraformaldehyde for 24 hours, and embedded in paraffin. Lylene was used to dewax, and gradient ethanol was used to hydration for 1 hour, then washed with distilled water. Antigen was repaired by boilingwater with 0.01M citric acid repair fluid for 15 minutes, wasded with PBS twice (15 minutes/time), and then blocked with serum for 20 minutes. Tissue sections were incubated with primary antibodies at room temperature for 2 hours, washed in PBST solution for three times. DAPI was used for nuclear staining. Tissue slides were incubated with fluorescein isothiocyanate (FITC) or phycoerythrin (PE)-conjugated secondary antibodies (Sangon, Shanghai) after primary antibody incubation. Immunofluorescence images were acquired using a fluorescent microscope. To visualize molecular markers of these markers, the IHC assay based on the horseradish peroxidase system was performed using specific antibodies. Mouse pancreatic tissues were sliced into piece with the thickness of 5 μm. Slides were incubated with antibodies against F4/80 (Bioss; diluted 1: 100), CD86 (Proteintech; diluted 1: 100), TLR2, (Signalway Antibody; diluted 1: 100), CD206 (Bioss; diluted 1: 100), ARG1 (Abclonal; diluted 1: 200) overnight at 4℃，and then were applied for staining and incubation by secondary antibodies at room temperature for 60 minutes. Positive staining of cells were separately evaluated by two experienced pathologists in a double-blinded manner. Criteria for histopathological scoring were: (1) 0, no positively stained cells; (2) 1, up to 25% positively stained cells; (3) 2, 26%–50% positively stained cells; (4) 3, 51%-75% positively stained cells, and (5) 4, >75% positively stained cells.The staining intensity was score as follow:0,negative;1,weak;2,moderate;and 3,strong. The final score was defined as staining number score multiplied by staining color score.

**Histological and fibrosis evaluation.**

Hematoxylin and Eosin Staining was performed to observe structural modification of tissues. The pancreatic tissues were stabilized with 4% paraformaldehyde for 24 hours to make a paraffin-embedded slice (embedded in paraffin. After fixation, the tissue was cut into 5 μm thick sections, and then stained with hematoxylin and eosin.). Tissue sections were dewaxed in xylene 2 times (5 minutes/time), dehydrated with gradient alcohol, and washed with distilled water (5 minutes). Then, slices were stained with hematoxylin (5 minutes) and differentiated with 1% hydrochloric acid (30 seconds), followed by 1% eosin-alcohol dyeing (5 minutes). Pancreatic sections were assessed under a microscope (magnification, 200×) over ten separate fields for severity of pancreatitis by scoring for edema, inflammatory infiltrate, acinar cell necrosis, and hemorrhage. Masson’s trichrome staining was to evaluate α-SMA and Collagen content in pancreatic tissue by Masson’s trichrome pancreatic kit according to manufacturer's specifications.

**Western blotting**

Total protein was extracted from the mouse pancreatic tissues using the Tissue or Cell Total Protein Extraction Kit (C510003; Sangon, Shanghai, China) following the manufacturer’s protocol. After protein concentration finished, protein from each sample was separated and transferred onto a PVDF membrane (Millipore, USA). The membrane was blocked with 5% non-fat milk for 2 hours at room temperature or 4°C overnight incubated with primary antibody, Finally, the membrane was incubated with horseradish peroxidase conjugated secondary antibody for 2 hours in the antibody buffer. GAPDH was used as the internal standard. Primary antibodies used in this study include anti-Atp8b1 (Abcam, 1:400), anti-Bhlha15 (Proteintech, 1:400), and anti-GAPDH (Signalway Antibody, 1:1000). The protein abundance was evaluated by immunoblotting with at least three biological replicates.

**Quantitative real-time PCR (qRT-PCR)**

qRT-PCR was carried out to detected mRNA expression in pancreatic tissues. Total RNA was extracted from samples using TRIzol reagent (Thermo Fisher Scientific, USA) compiled with the manufacturer’s protocol. About 3.0 μg of total RNA was converted to cDNA using the High Capacity cDNA Reverse Transcription Kit (Thermo Fisher Scientific, USA) as suggested by the manufacturer’s protocol. Relative gene mRNA expression was evaluated by quantitative RT-PCR using a QuantiFast SYBR Green PCR Kit (Qiagen, German) following the manufacturer’s instructions. All assay was performed at least in triplicate. The sequences of the primers used are listed in Supplementary Table S1.

**Enzyme-Linked Immunosorbent Assay (ELISA)**

Supernatants from mice pancreatic tissues were collected and stored at -80℃ after removal of serum by centrifugation. The protein concentration of IL-1β, IL-6, and TNF-α in the serum were detected using specific ELISA kits (Signalway Antibody) according to the manufacturer’s instruction.

**ATAC‑seq**

Pancreatic tissues from *PRSS1^Tg^* mice treated with caerulein for 4 weeks (Group B) and 1 week (Group A) were cut into small pieces and added 10mL 200U/mL collagenase IA solution for digestion at 37°C for 25 minutes, then the cells were blown out and planted in a type I collagen-coated culture dish and the pancreatic cells were collected and counted. In accordance with the ATAC-seq protocol, approximately 50,000 cells were used for cell lysis (10mM Tris-Hcl, pH=7.4, 10mM Nacl, 3mM Mgcl_2_, 0.1%IGEPAL CA-630) and precipited with centrifugation at 500g for 10 minutes. The nuclei pellet was resuspended in 50 μL transposition reaction mixture which includes 25 μL Tagment DNA buffer (TruePrep DNA Library Prep Kit V2 for Illumina, vazyme), 2.5 μL Tn5 transposase (TruePrep DNA Library Prep Kit V2 for Illumina, vazyme), and 22.5μL nuclease-free water, and incubated at 37 °C for 30 min. The DNA fragments were then purified using a MinElute PCR Purification Kit (Qiagen, Cat. No. 28004) and we amplified library fragments using 1× NEBnext PCR master mix and 1.25μM custom Nextera PCR primers, using the following PCR conditions: 72°C for 5 minutes; 98°C for 30 seconds; and thernocycling at 98°C for 10 seconds, 63°C for 30 seconds and 72°C for 1 minutes. We first amplified the full libraries for 5 cycles and then amplified 8 cycles. Amplified library was future purified with 0.6× and 1.5× volume of pure beads (KK8002, KAPA). The ATAC-seq libraries were quantified using the DNA High Sensitivity kit (Agilent) on a Bioanalyzer. The sequencing was performed on an Illumina NovaSeq 6000. we used the FASTQC pipeline (Version 0.3.2) on our reads, and aligned them to the reference genome (mm10) bowtie2 (version 2.3.3.1) for ATAC-seq, retaining only reads that mapped to a unique position in the genome. (Version 0.3.2).

**ChIP‑seq**

Pancreatic tissues from *PRSS1^Tg^* mice treated with caerulein for 4 weeks (Group B) and 1 week (Group A) were cut into small pieces and added 10mL 200U/mL collagenase IA solution for digestion at 37°C for 25 minutes, then the cells were blown out and planted in a type I collagen-coated culture dish and the pancreatic cells were collected and counted. In accordance with the ATAC-seq protocol, approximately 1 × 10^7^ cells were cross-linked in 1% formaldehyde for 10 minutes at room temperature, and the reaction was then quenched with 125 mM glycine for 5 minutes. After lysis in 0.5% Igepal CA-630 in PBS supplemented with protease inhibitors for 10 minutes, nuclei were pelleted at 6000g and digested with 1 μl of micrococcal nuclease for 3 minutes at 37 °C to fragment chromatin. Next, then resuspended in SDS lysis buffer (1% SDS, 10mM EDTA, 50mM Tris-HCl, pH 8.1) at a ratio of 1mL per 2×10^7^ cells. Pulse sonications were performed for 9 minutes at 40% amplitude with 30% on/70% off on a Brandon Digital Sonifier (Model 250) with a total of 1 mL with maximum 50% SDS Lysis Buffer solution diluted with ChIP Dilution Buffer (0.01%SDS, 1.1% Triton X-100, 1.2mM EDTA, 16.7mM Tris-HCl, pH8.1, 167mM NaCl). ChIPs were performed with antibodies for H3K27me3 (Abcam, ab192985) and RNA ploymerase II (Abcam, ab264350). Pulldowns were performed with Dynabeads Protein A/G (Abcam, ab286842). DNA quantities were measured by Qubit 3.0 (Qubit™ 1X dsDNA Assay Kits, high sensitivity (HS) and broad range, Q33230) and Bioanalyzer. ChIP-seq libraries were prepared using the Illumina TruSeq ChIP Library Preparation Kit. The sequencing was performed on an Illumina NovaSeq6000. The reads were aligned to a mouse genome (mm10) using Bowtie2 (version 2.3.3.1) with default parameters. Duplicate reads were removed using SAMtools (v 1.2), Peaks were called using MACS2. DiffBind was used to analyse differential peaks.

**References:**

1 Guo P, Wiersch J, Xiao X, Gittes G. Simplified Purification of AAV and Delivery to the Pancreas by Intraductal Administration. *Methods Mol Biol* 2019;1950（:373-387.
