# Supplementary material for: Acinar ATP8b1/LPC pathway promotes macrophage efferocytosis and clearance of inflammation during chronic pancreatitis development
Source: Cell Death Dis. 2022 Oct 22;13(10):893. doi: 10.1038/s41419-022-05322-6 (PMC9588032; doi:10.1038/s41419-022-05322-6)
Supplement: Supplementary file 9 — Table S1 [file 41419_2022_5322_MOESM9_ESM.docx]

**Table S1. Primer sequences used for quantitative RT-PCR assay.**

| **Primer ID** | **Sequences (5’-3’)** |
| --- | --- |
| **Bhlha15 F** | GACATGTGACGCCTCAGGAA |
| **Bhlha15 R** | GGGGAAAAATAGCGGGTCCA |
| **Atp8b1 F** | CAATGGCTACTCAGCCCAGA |
| **Atp8b1 R** | TCAGCTTGTCACTCACGTCC |
| **CD206 F** | CAGGAGGACTGCGTGGTTATG |
| **CD206 R** | GGTTTGCATCAGTGAAGGTGG |
| **ARG1 F** | CTGGGGATTGGCAAGGTGAT |
| **ARG1 R** | CAGCCCGTCGACATCAAAG |
